# Combined Targeting of NAD Biosynthesis and the NAD-dependent Transcription Factor C-terminal Binding Protein as a Promising Novel Therapy for Pancreatic Cancer

**DOI:** 10.1158/2767-9764.CRC-22-0521

**Published:** 2023-10-04

**Authors:** M. Michael Dcona, Kranthi Kumar Chougoni, Diana T. Dcona, Jacqueline L. West, Sahib J. Singh, Keith C. Ellis, Steven R. Grossman

**Affiliations:** 1 USC Norris Comprehensive Cancer Center and Department of Medicine, Keck School of Medicine, University of Southern California, Los Angeles, California.; 2 Department of Medicinal Chemistry and Institute for Structural Biology, Drug Discovery and Development, Virginia Commonwealth University, Richmond, Virginia.; 3 VCU Massey Cancer Center, Virginia Commonwealth University, Richmond, Virginia.

## Abstract

Cancer therapies targeting metabolic derangements unique to cancer cells are emerging as a key strategy to address refractory solid tumors such as pancreatic ductal adenocarcinomas (PDAC) that exhibit resistance to extreme nutrient deprivation in the tumor microenvironment. Nicotinamide adenine dinucleotide (NAD) participates in multiple metabolic pathways and nicotinamide phosphoribosyl transferase (NAMPT) is one of the key intracellular enzymes that facilitate the synthesis of NAD. C-terminal binding proteins 1 and 2 (CtBP) are paralogous NAD-dependent oncogenic transcription factors and dehydrogenases that nucleate an epigenetic complex regulating a cohort of genes responsible for cancer proliferation and metastasis. As adequate intracellular NAD is required for CtBP to oligomerize and execute its oncogenic transcriptional coregulatory activities, we hypothesized that NAD depletion would synergize with CtBP inhibition, improving cell inhibitory efficacy. Indeed, depletion of cellular NAD via the NAMPT inhibitor GMX1778 enhanced growth inhibition induced by either RNAi-mediated CtBP1/2 knockdown or the CtBP dehydrogenase inhibitor 4-chlorophenyl-2-hydroxyimino propanoic acid as much as 10-fold in PDAC cells, while untransformed pancreatic ductal cells were unaffected. The growth inhibitory effects of the NAMPT/CtBP inhibitor combination correlated pharmacodynamically with on-target disruption of CtBP1/2 dimerization, CtBP2 interaction with the CoREST epigenetic regulator, and transcriptional activation of the oncogenic target gene TIAM1. Moreover, this same therapeutic combination strongly attenuated growth of PDAC cell line xenografts in immunodeficient mice, with no observable toxicity. Collectively, our data demonstrate that targeting CtBP in combination with NAD depletion represents a promising therapeutic strategy for PDAC.

**Significance:**

Effective precision therapies are lacking in PDAC. We demonstrate that simultaneous inhibition of NAD metabolism and the oncoprotein CtBP is potently effective at blocking growth of both PDAC cells in culture and human PDAC-derived tumors in mice and should be explored further as a potential therapy for patients with PDAC.

## Introduction

Pancreatic ductal adenocarcinoma (PDAC) remains among the most lethal of human cancers with a 5-year survival rate of approximately 10% (1). Recent identification of low-frequency druggable genomic alterations in PDAC has contributed only minimally to improved survival (1). PDAC exhibits a poorly vascularized desmoplastic stroma, leading to a hypoxic and nutrient-deficient environment (2), hence targeting metabolic dependencies in PDAC provides an opportunity to develop novel PDAC therapeutics to address the overarching problem of inherent and acquired therapeutic resistance.

Nicotinamide adenine dinucleotide (NAD) is a frequent metabolic dependency in solid tumors (3). NAD metabolism is regulated at the level of both synthesis and consumption, and a decrease in cellular NAD levels can lead to metabolic collapse and cell death. These unique features of NAD metabolism in cancer cells, in turn, have opened new avenues for therapeutics that target NAD synthesis and/or consumption, representing a promising and novel therapeutic modality for a variety of human solid tumors, including PDAC.

**FIGURE 1:**
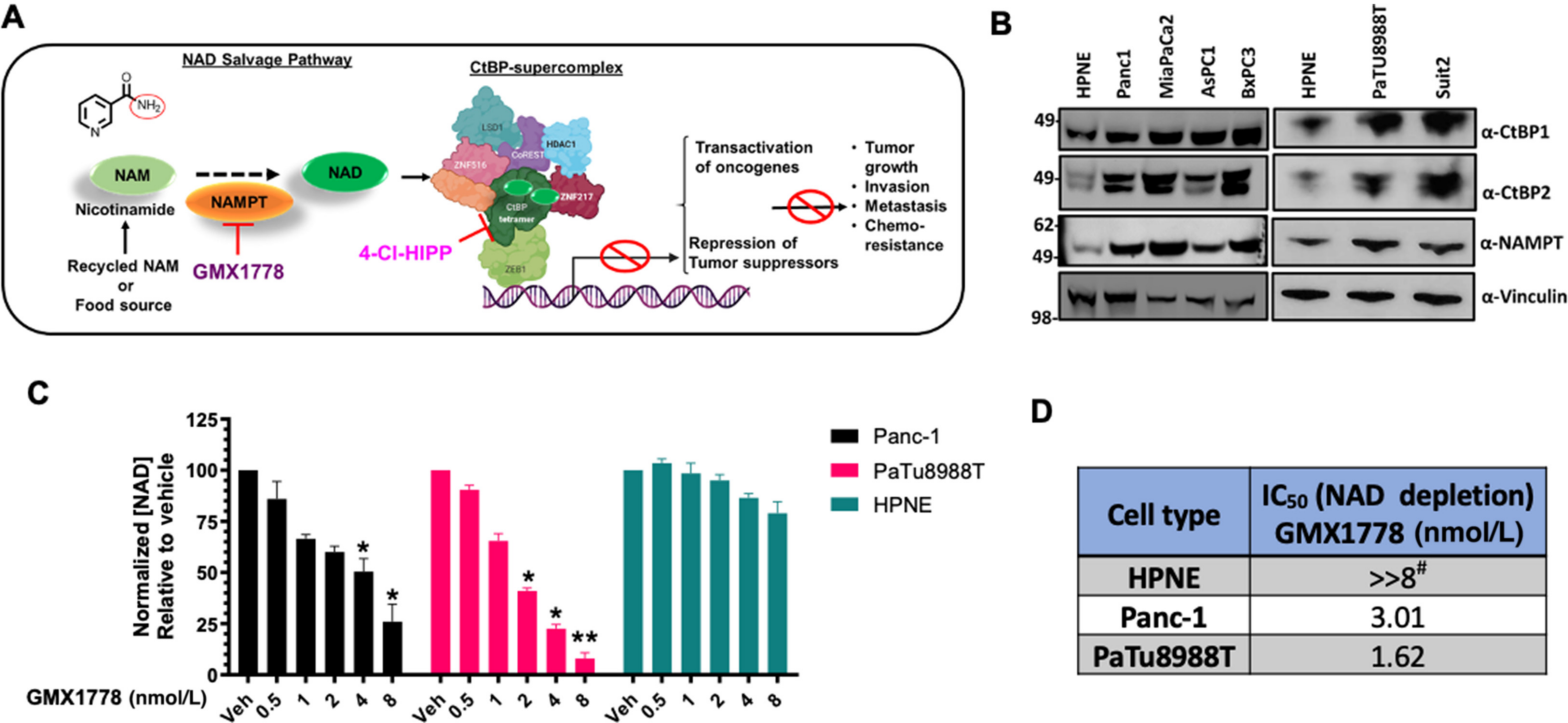
NAMPT is highly expressed and regulates NAD in PDAC cells. **A,** Schematic illustrating the intersection of NAD biosynthesis and CtBP protein–protein interactions and related oncogenic transcriptional function. **B,** Immunoblot analysis of CtBP1/2 and NAMPT in hTERT-HPNE (HPNE) and indicated PDAC cell lines. Vinculin served as loading control. Representative immunoblots shown; *N* = 3 independent experiments. **C,** Quantification of total NAD [NAD(H) + NAD^+^] in Panc-1, PaTu8988T, and HPNE cells after treatment with Vehicle (Veh) or increasing concentrations of GMX1778 for 24 hours, using commercial assay kit (Sigma-Aldrich). Values represent the percent change in total NAD concentration relative to vehicle (Veh) treatment for each cell line. *P* values were determined by Student *t* test. **D,** GMX1778 IC_50_ values for half-maximal reduction of total cellular NAD concentration in the indicated cell lines. ^#^indicates the IC_50_ could not be calculated and beyond the upper range of the titration. Error bars indicate the SEM. *N* = 3 independent experiments. *, *P* < 0.05; **, *P* < 0.01 for comparison with vehicle-only treatment.

In mammalian cells, NAD is synthesized via three major pathways, namely, the *de novo*, Preiss-Handler (PH), and salvage pathways with tryptophan, nicotinic acid (NA), and nicotinamide (NAM), as their precursors, respectively (Fig. 1A; Supplementary Fig. S1; ref. 4). Cancer cells have little to no ability to use the *de novo* pathway to generate NAD, and thus rely on either or both the salvage and PH pathways for generation of NAD (5). Nicotinamide phosphoribosyl transferase (NAMPT) is the apical and rate-limiting enzyme of the salvage pathway (Fig. 1A; Supplementary Fig. S1) and has been pursued as a cancer therapeutic target, as it is often overexpressed in cancer, and certain tumors exhibit absolute dependency on the salvage pathway due to lack of functional *de novo* or PH pathways (4, 5). However, NAMPT inhibitors (NAMPTi) have exhibited limited success in the clinic, due to both lack of robust efficacy as single agents, as well as toxicities, which include dose-limiting thrombocytopenia (5).

C-terminal binding proteins 1 and 2 (CtBP1/2; hereafter “CtBP”—as they are paralogous genes with overlapping function) are oncogenic transcriptional coregulators that are overexpressed in many solid cancers, and their expression universally correlates with worse prognosis (6). Unique among eukaryotic transcription factors, CtBP encodes a fully functional dehydrogenase domain that utilizes nicotinamide adenine dinucleotide hydride (NADH) as a hydrogen donor cofactor to convert a methionine intermediate metabolite, methyl-thio-oxybutyric acid (MTOB), to the hydroxy form (MTHB; ref. 7). Notably, CtBP's ternary structure (8), its interaction with other epigenetic regulatory proteins (9), and its coregulatory transcriptional functions (6, 10) are highly dependent upon levels of NAD (both NAD+/NADH; Fig. 1A; ref. 11). CtBP and its interactome, which includes class 1 histone deacetylases, demethylases (e.g., KDM1), and other chromatin modifiers, contribute to malignant progression by corepressing proapoptotic and tumor-suppressing genes, and coactivating oncogenes (Fig. 1A; refs. 6, 12–14).

Validating its oncogenic functions in mouse models, *Ctbp2* haploinsufficiency dramatically reduced intestinal polyposis and prolonged survival in the *Apc^min^* intestinal polyposis model and also extended survival and attenuated peritoneal metastasis in a mutant K-Ras–driven mouse PDAC model (13). Given CtBP's robust validation as a cancer therapeutic target in multiple *in vivo* systems, we have developed a library of CtBP dehydrogenase substrate competitive inhibitors (CtBPi) in an effort to therapeutically inactivate CtBP's oncogenic functions. Our lead CtBPi, 4-chlorophenyl-2-hydroxyimino propanoic acid (4-Cl-HIPP; ref. 15) phenocopied allelic *Ctbp2* loss in *Apc^min^* mice, including extension of survival (13), albeit at dosages that warrant further optimization or combination with another agent that can amplify its effective potency.

In this study, we have investigated the effect of inhibiting NAMPT-regulated salvage pathway NAD biosynthesis in PDAC cells and tumors using the NAMPTi tool compound GMX1778 as a means of sensitizing PDAC cells to genetic or pharmacologic inhibition of CtBP. We demonstrate that GMX1778 dramatically increased 4-Cl-HIPP effectiveness, decreasing its half-maximal growth inhibitory concentration (GI_50_) for PDAC cell lines 5- to 10-fold. The combination of NAMPT and CtBP inhibitors also cooperatively modulated CtBP's coregulatory activities and effectively attenuated tumor growth in a mouse xenograft model. Complementing and validating the pharmacologic effects of combined NAMPT/CtBP inhibition, CtBP depletion via RNAi in combination with NAMPTi mimicked the effects of inhibitor combination treatments. We therefore propose that the pharmacologic perturbation of NAD synthesis in PDAC cells attenuates CtBP's oncogenic activity and renders cells highly sensitive to CtBP dehydrogenase inhibitors. Taken together, our data support combined pharmacologic inhibition of NAMPT and CtBP as a novel and effective therapeutic strategy in PDAC worthy of further investigation.

## Materials and Methods

### Cell Culture

Panc-1 (RRID: CVCL_0480), MiaPaCa2 (RRID: CVCL_0428), AsPc1 (RRID: CVCL_0152), BxPC3 (RRID: CVCL_0186), and human telomerase reverse transcriptase (hTERT)-HPNE (RRID: CVCL_C466) cell lines were purchased from ATCC. PaTu8988T (RRID: CVCL_1847) and SUIT2 (RRID: CVCL_3172; ref. 16) were kind gifts from T. Donahue [University of California Los Angeles (UCLA)] and were verified by short tandem repeat analysis (University of Arizona Genetics Core). All PDAC cell lines were maintained as per ATCC recommendations in RPMI1640, DMEM, or McCoy's media supplemented with 10% (v/v) FBS and 1% penicillin-streptomycin, unless otherwise specified. hTERT-HPNE cells were maintained in a base medium that comprised 75% DMEM without glucose (with an additional 2 mmol/L l-glutamine and 1.5 g/L sodium bicarbonate), 25% Medium M3 Base (Incell Corp. #M300F-500), 5% FBS, 10 ng/mL human recombinant EGF, 5.5 mmol/L d-glucose (1 g/L), and 750 ng/mL puromycin. HEK-293T cells used to produce lentivirus were cultured in DMEM supplemented with 10% (v/v) FBS and penicillin-streptomycin. All cells were used for experiments within 3 months of thawing, maintained in a humidified incubator equilibrated with 5% CO_2_ at 37°C, authenticated by examination of morphology and growth characteristics, and were confirmed to be *Mycoplasma* free (last tested June 13, 2023) using a PCR-based kit (Abcam).

### Antibodies and Immunoblot Analyses

Antibodies used for immunoblotting and immunoprecipitation (IP) were: CtBP2 (Santa Cruz Biotechnology, discontinued; E-16/sc-5966); CtBP1 (BD Biosciences, #612042; RRID:AB_399429); CtBP1 (Santa Cruz Biotechnology, discontinued; G-6/sc-5963; RRID:AB_2086637); NAMPT (Santa Cruz Biotechnology, E-3/sc-393444); NAPRT (Santa Cruz Biotechnology, B-8/sc-398404); CtBP2 (Cell Signaling Technology, #13256; RRID:AB_2798164); Vinculin (Cell Signaling Technology, E1E9V mAb #13901; RRID:AB_2728768); CoREST (Bethyl, #A300-130A; RRID:AB_242523); GAPDH (Santa Cruz Biotechnology, sc-32233; RRID:AB_627679). For immunoblot analysis, 10–30 µg of total protein extract was boiled at 95°C in 1X NuPage LDS Sample Buffer (Novex) followed by separation on SDS-PAGE, and then transferred onto nitrocellulose membrane (0.45 µm; GE Healthcare). The membrane was incubated for 1 to 2 hours in blocking buffer (50 mmol/L Tris pH 7.6, 150 mmol/L NaCl, 0.1% Tween-20, [TBS-T] + 5% nonfat dry milk), followed by incubation overnight at 4°C with the primary antibody (1:1,000) diluted in blocking buffer with sodium azide (0.01%). After three washes of 5 minutes each in TBS-T, blots were incubated with fluorescently labeled or horseradish peroxidase-conjugated anti-rabbit or anti-mouse secondary antibodies for 1 hour in TBS-T/1% nonfat dry milk and visualized on Odyssey (LI-COR) or Chemi-Doc (Bio-Rad) imagers, respectively. Secondary antibodies used were: anti-rabbit Alexa Fluor 680 (1:3000; Invitrogen, #A-21076; RRID AB_2535736); anti-mouse Alexa Fluor 790 (1:3000; Invitrogen, #A11375; RRID AB_2534146); anti-rabbit HRP (1:5000; Cell Signaling Technology, #7074); anti-mouse HRP (1:5000; Cell Signaling Technology, #7076).

### Pharmacologic Inhibitors

4-Cl-HIPP was synthesized as described previously (15) and prepared as a 50 mmol/L stock solution in 100 mmol/L NaHCO_3_ (pH = 8.5) and stored at −20°C. GMX1778 (Selleckchem #S8117) was solubilized in DMSO as a 50 mmol/L stock solution and stored at −20°C. 100 mmol/L NaHCO_3_ was used as the vehicle for all experiments.

### Cell Viability (MTT) Assay

A total of 3,000–5,000 PaTu8988T or SUIT2 cells expressing shGFP, shCtBP1 or shCtBP2 were seeded into a 96-well plate (Corning) in triplicate and allowed to grow for 24 hours. Vehicle or increasing concentrations of GMX1778 were then added and cells were incubated for an additional 72 hours. 3-(4,5-dimethylthiazol-2-yl)-2,5-diphenyltetrazolium bromide (MTT; 0.5 g/mL in DMEM; Invitrogen, #M6494) was then added to each well and the plate incubated for an additional 4 hours. The MTT solution was then removed and replaced with 100% DMSO to dissolve the formazan crystals and the A_570_ nm of each well determined using a microplate reader (Clariostar). The A_570_ values were then used to calculate the percentage of viable cells in each well relative to vehicle-treated cells.

### Colony Formation Assay

Cells were seeded at 50–200 cells/well in triplicate in 6-well plates in 2 mL media per well. After overnight incubation, vehicle or GMX1778 was added for 24 hours, followed by addition of vehicle or 4-Cl-HIPP for an additional 5 days (Panc-1) or 7 days (HPNE, Patu8988T, BxPC3, and AsPC1). Colonies were then fixed using cold methanol (100%) and stained with 0.5% crystal violet in 50% methanol for 10 minutes, washed in 50% methanol, dried, and quantitated by either ImageJ (NIH) densitometric analysis or by solubilizing the stained colonies in DMSO and then measuring the absorbance at 592 nm.

### NAD/NADH Quantification

After drug treatment of PaTu8988T, Panc-1, or HPNE cells in 6-well plates for 24 hours, harvested cell pellets were centrifuged at 2,000 revolutions per minute (RPM) and incubated with 400 µL of NADH/NAD extraction buffer by freeze/thawing for two cycles of 20 minutes on dry ice, followed by 10 minutes at room temperature. Samples were then vortexed for 10 seconds, centrifuged at 13,000 × *g* for 10 minutes to remove insoluble material, and the supernatant was assayed for NAD/NADH concentration using a commercial assay (Sigma-Aldrich, #MAK037) per manufacturer's instructions.

### *In Vivo* Cross-linking

Disuccinimidyl glutarate (DSG) cross-linker (Thermo Fisher Scientific, #20593) was prepared as a stock solution of 10 or 50 mmol/L in DMSO made fresh for each experiment. *In vivo* cross-linking was carried out in Panc-1 or PaTu8988T cells that were collected by scraping, washed with cold PBS (pH 7.2), and resuspended in PBS (pH 8.2) with 1X Complete Protease Inhibitor Mixture, EDTA-free (Roche). Samples were incubated with cross-linker (1 mmol/L) for 30 minutes at 37°C with rotation. The reaction was quenched with the addition of 1 mol/L Tris HCl pH 7.6 to 20 mmol/L final concentration and incubated for 15 minutes at room temperature. After quenching, *in vivo* cross-linked samples were lysed using RIPA Lysis and Extraction Buffer (Thermo Fisher Scientific, #89900) followed by ultracentrifugation for 30 minutes at 15,000 rpm at 4°C. Samples were then immunoblotted for CtBP1 or CtBP2.

### IPs

After treatment with relevant drug conditions, Panc-1 or PaTu8988T cells were lysed using IP buffer (20 mmol/L Tris, pH 7.5, 150 mmol/L NaCl, 1% NP40, protease inhibitor tablet-added fresh). Whole-cell lysates (1.0 mg) were precleared by incubation with protein G Agarose Fast Flow beads (EMD Millipore) for 1 hour on a rotator at 4°C. A total of 2 µg of CtBP1 or CtBP2 antibody was then added to the precleared lysates and incubated on a rotator at 4°C for 1 hour. Washed protein G Agarose Fast Flow beads were then added and incubated overnight, followed by three washes in IP buffer and resuspension of the washed beads in 40 µL of 1X LDS NuPage buffer, and heating to 95°C for 10 minutes. After centrifugation of the beads, supernatants were loaded on SDS-PAGE, followed by immunoblotting.

### qPCR

After relevant treatments, cellular mRNAs were isolated from samples using QIAGEN RNeasy kit. cDNA synthesis was then carried out using SensiFAST cDNA Synthesis Kit (BIOLINE). Quantitation of all gene transcripts was done by quantitative PCR (qPCR) using SYBR Green (Applied Biosystems) and an ABI 7300 (Applied Biosystems) PCR machine. 18S rRNA was used as an internal control. The primer pairs used were as follows:

**18S rRNA**
  - 5′-CGCCGCTAGAGGTGAAATTC-3′(forward) and
  - 5′-TGGCAAATGCTTTCGCTCTG-3′ (reverse).
**TIAM1**
  - 5′-CGCTGGAGTCGTACCTCATC-3′ (forward) and
  - 5′-GGTCAAACACAGCCCCAAAC-3′ (reverse).

Relative amounts of the mRNA transcripts were calculated using the ΔΔCt method and reported as fold change with respect to vehicle control (17).

### Lentiviral Short Hairpin RNA

CtBP1 (TRCN0000273842) and CtBP2 (TRCN0000013744) short hairpin RNA (shRNA)-expressing lentiviruses (Mission shRNA-Millipore) were produced in packaging cells (HEK293T; RRID: CVCL_0063) by cotransfection of appropriate transfer vector (Sigma-Aldrich) with compatible packaging plasmid (pCMV-deltaR8.1; RRID: Addgene_12263) and envelope vector (VSV-G; RRID: Addgene_138479) using Lipofectamine 2000 Transfection Reagent (Invitrogen). PDAC cells were the incubated with the viral particles and selected with puromycin as described previously (18).

### Migration Assay

PaTu8988T cells were seeded into a glass-bottom 35 mm dish (Nunc, **#**150680) at a density of 2.5 × 10^4^ cells/dish in 0.5 mL. Cells were then treated with vehicle (100 mmol/L NaHCO_3_) or 2 nmol/L GMX1778 for 24 hours followed by addition of vehicle or 250 µmol/L of 4-Cl-HIPP for 48 hours. Cells were then washed, incubated with 2 mL of ice-cold methanol for 15 minutes, and then stained with 0.5% crystal violet in 50% methanol for 10 minutes, washed in 50% methanol, dried, and quantified using ImageJ.

### Mouse Xenografts

All animal experiments were performed with approval and supervision of the USC Institutional Animal Care and Use Committee. Panc-1 cells were trypsinized, checked for >95% viability by trypan blue assay and resuspended in 50% PBS + 50% phenol red–free Matrigel at a final concentration of 1 × 10^7^ cells/mL. A total of 100 µL of cell suspension (1 × 10^6^ Panc-1 cells) was then subcutaneously injected into the right flank of each mouse and after 2 weeks of tumor growth, xenografted mice were treated three times per week over 4 weeks (days 0–28) by intraperitoneal injection of vehicle (100 mmol/L NaHCO_3_), 4-Cl-HIPP (100 mg/kg in vehicle), GMX1778 (30 mg/kg in vehicle), or the combination of 4-Cl-HIPP and GMX1778. Tumor volume and mouse weights were determined weekly during treatment and at termination of the experiment on day 28. On day 28, mice were euthanized, and tumor weights were determined from necropsied tumors.

### Statistical Analysis

Statistical comparisons were performed utilizing Student *t* test, multiple unpaired Student *t* test or one-way ANOVA using Tukey *post hoc* correction as indicated, with the threshold for statistical significance set at *P* < 0.05 (19).

### Data Availability Statement

The data generated in this study are available upon request from the corresponding author.

## Results

### NAMPT and CtBP1/2 Protein Abundances are Elevated in PDAC Cell Lines

On the basis of CtBP's key role in pancreatic tumorigenesis (13) and the coordinated requirement of NAD for CtBP's oncogenic activities (11, 20), we investigated whether there were discernable differences in the levels of CtBP1/2 and the key NAD biosynthetic enzyme NAMPT between an untransformed pancreatic cell line and PDAC cell lines. To this end, we compared NAMPT and CtBP1/2 protein abundances between a set of six PDAC cell lines—Panc-1, MiaPaCa-2, AsPc-1, BxPC-3, PaTu8988T, and SUIT2—that each harbor distinctive KRAS and TP53 genetic alterations (Supplementary Table S1), and the “normal” (nontransformed but immortalized) pancreatic reference cell line hTERT-HPNE (HPNE), which is derived from non-neoplastic human pancreas cells stably transduced with human telomerase reverse transcriptase (hTERT; Fig. 1B; ref. 21). Immunoblotting of lysates from each of these cell lines revealed that levels of CtBP1/2 and NAMPT were higher in PDAC cell lines compared with HPNE cells, suggesting that both CtBP and NAMPT enzymatic activities may be hyperactive in PDAC tumor cells compared with normal pancreatic ductal cells (Fig. 1B; Supplementary Fig. S2).

### Treatment of PDAC Cells with the NAMPTi GMX1778 Decreases Total NAD Levels

As CtBP activity is directly correlated with the stoichiometric availability of cellular NAD (11), we determined whether NAMPT inhibition, which has been used as a strategy for cellular NAD depletion in a number of tumor types (5, 22, 23), can effectively lower NAD levels in PDAC cells. We therefore treated PaTu8988T, Panc-1, or HPNE cells with increasing concentrations of the potent NAMPTi tool compound GMX1778 (22) for 24 hours, followed by assay for intracellular levels of total NAD species (Fig. 1C). Consistent with prior reports (22, 23), increasing doses of GMX1778 significantly decreased the levels of total NAD in PaTu8988T and Panc-1 cells by 3- to 4-fold, while barely impacting total NAD levels in HPNE cells (Fig. 1C), possibly due to the availability of alternative NAD biosynthetic pathways in nontransformed HPNE cells, allowing them to maintain stable NAD homeostasis in the face of inhibition of the NAMPT-dependent salvage pathway (4). Indeed, the GMX1778 IC_50_ values for NAD depletion in the PDAC cell lines were in the low nanomolar range, while NAD levels in HPNE cells decreased only approximately 20% at the maximal GMX1778 concentration tested of 8 nmol/L (Fig. 1D).

### CtBP Depletion Sensitizes PDAC Cells to Growth Inhibitory Effects of NAMPT Inhibition

**FIGURE 2:**
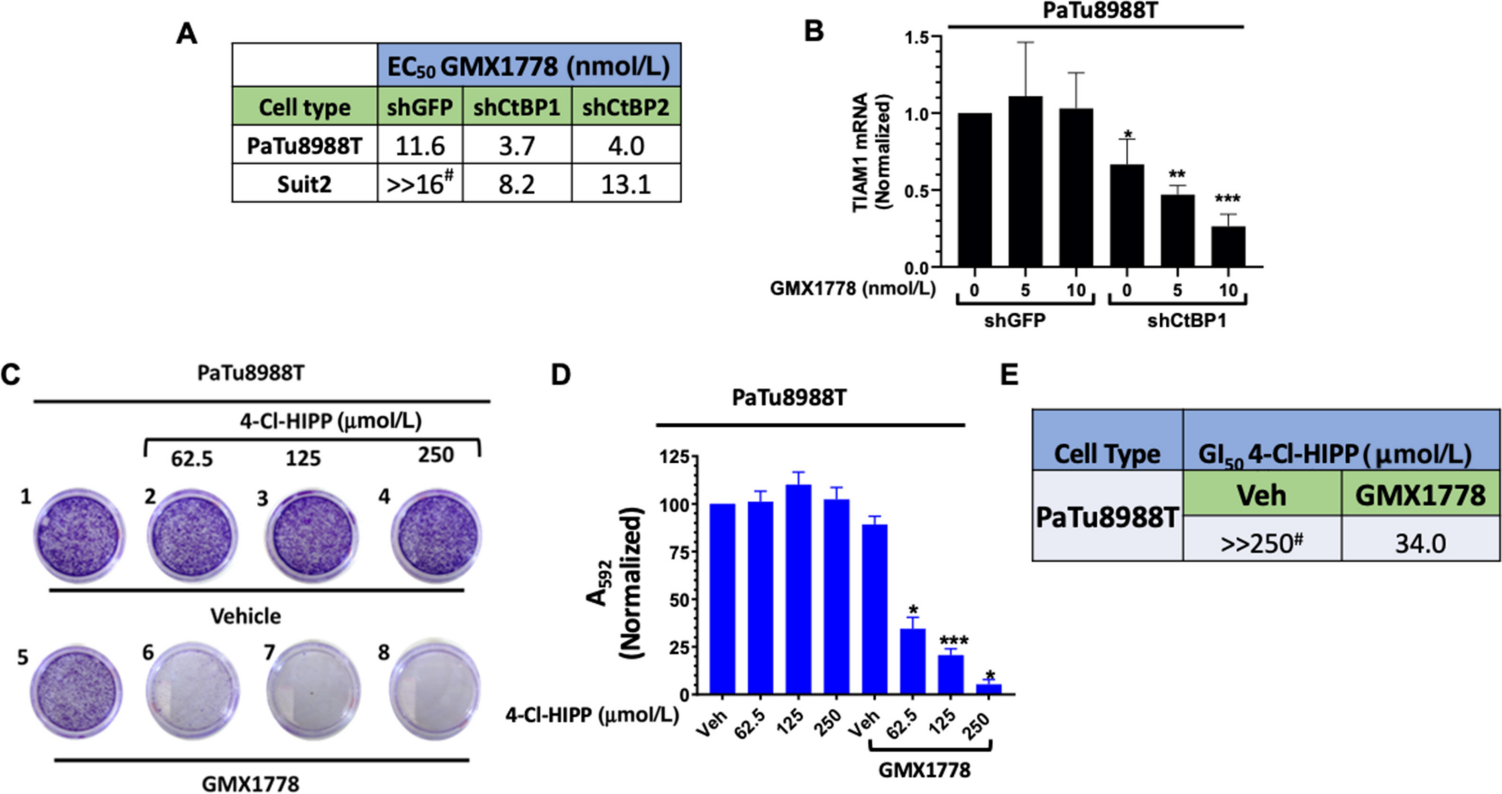
Genetic depletion or pharmacologic inhibition of CtBP sensitizes PDAC cells to GMX1778 growth inhibition. **A,** GMX1778 EC_50_ values for PaTu8988T or SUIT2 cells expressing shGFP, shCtBP1, or shCtBP2 as measured by 72 hours MTT assay. **B,** qPCR analysis of *TIAM1* (CtBP target gene) mRNA expression after treatment of PaTu8988T cells expressing shGFP or shCtBP1 with Vehicle (0) or indicated concentrations of GMX1778 for 24 hours. The calculation was performed using the ΔΔCt approach. *P* values were determined by Student *t* test for comparison with vehicle-treated shGFP cells. **C,** Representative colony formation assay of PaTu8988T cells treated with Vehicle (top row) or 2 nmol/L GMX1778 (bottom row) for 24 hours, followed by treatment with Vehicle (plate 5) or indicated concentrations of 4-Cl-HIPP (plates 6–8) for 7 days, followed by crystal violet staining. **D,** Colony formation assay in C was quantified by measuring A_592_ of solubilized crystal violet. *P* values were determined by Student *t* test for comparison with vehicle-only treated cells. **E,** Calculation of GI_50_ values for 4-Cl-HIPP treatment of PaTu8988T cells with or without 2 nmol/L GMX1778 derived from colony formation assay in C. ^#^indicates the EC_50_ or GI_50_ could not be calculated and beyond the upper range of the titration of GMX1778 or 4-Cl-HIPP, respectively. Error bars indicate ± 1 SD. *N* = 3 independent experiments. *, *P* < 0.05; **, *P* < 0.01; ***, *P* < 0.005.

Having demonstrated that NAMPTi could effectively lower cellular NAD levels in PDAC cells, we next tested the hypothesis that lowering cellular NAD levels via NAMPTi would sensitize PDAC cells to genetic and subsequently, pharmacologic, inactivation of CtBP. For this purpose, we assayed the cell viability of PaTu8988T and SUIT2 cells expressing shCtBP1, shCtBP2 or control shGFP (Supplementary Fig. S3) treated with increasing doses of GMX1778 (Fig. 2A; Supplementary Fig. S4). Strikingly, compared with cells with shGFP expression, shCtBP1 or shCtBP2-expressing cells displayed significantly enhanced sensitivity toward GMX1778 treatment with PaTu8988T (shGFP) half-maximal effective concentration (EC_50_) = 11.5 versus 3.6 nmol/L (shCtBP1) or 3.9 nmol/L (shCtP2) and SUIT2 (shGFP) EC_50_ ≫16 nmol/L versus 8.2 nmol/L (shCtBP1) or 13.1 (shCtP2; Fig. 2A; Supplementary Fig. S4). Thus, CtBP1 or CtBP2 depletion cooperates with GMX1778-mediated NAMPT inhibition, driving at least a 1.5- to 5-fold enhancement of GMX1778 growth inhibitory potency. This proof of principle study employing a combination of NAMPTi and shRNA depletion of CtBP1/2 therefore predicts that combined pharmacologic inhibition of NAMPT and CtBP could be potently growth inhibitory in PDAC cells and tumors.

### NA Rescues PDAC Cells from the On-target Growth Inhibitory Effects of GMX1778

To verify that NAD depletion observed after GMX1778 treatment was due to on-target inhibition of NAMPT and the NAD salvage pathway, we inquired whether supplying the precursor for an alternative NAD synthetic pathway, such as the NAPRT-dependent PH pathway, would rescue PDAC cells from growth inhibition induced by GMX1778 alone (Supplementary Fig. S5). To that end, we determined the EC_50_ for GMX1778 in PaTu8988T cells pretreated with vehicle or the PH precursor NA (4), and indeed, NA strongly rescued the growth inhibitory effects of GMX1778 increasing the EC_50_ at least 6- to 10-fold (Supplementary Fig. S5).

### GMX1778 and CtBP Depletion Cooperatively Block Transactivation of the Oncogenic CtBP Transcriptional Target Gene TIAM1

To validate that the cell inhibitory effects of combined NAMPT/CtBP depletion reflected on-target inhibition of CtBP's transcriptional activities, we utilized qPCR analysis to quantify the amount of *TIAM1* mRNA in shGFP or shCtBP1 cells treated with vehicle, 5 or 10 nmol/L of GMX1778 for 24 hours (Fig. 2B). Tiam1 is a well-characterized transcriptional activation target of CtBP that drives tumor progression and metastasis in several cancer model systems (14, 24). As expected, shCtBP1-expressing cells exhibited an approximately 25% decrease of *TIAM1* mRNA levels relative to shGFP-expressing cells (10, 14), and addition of GMX1778 to shGFP-expressing cells had no effect (Fig. 2B). However, treatment of cells expressing shCtBP1 with GMX1778 led to further robust decrease in the levels of *TIAM1* mRNA in a GMX1778 dose-dependent manner (75% reduction in *TIAM1* level relative to vehicle treatment; Fig. 2B), indicating a strong cooperative effect of NAMPT and CtBP depletion that leads to on-target diminished CtBP-mediated transcriptional coregulatory activities.

### NAMPT Inhibition Sensitizes PDAC Cells Toward Pharmacologic CtBP Inhibition

We next investigated whether the cooperative antiproliferative effects of combining NAMPT inhibition with CtBP depletion via RNAi could be phenocopied by combining a small-molecule CtBP dehydrogenase inhibitor (4-Cl-HIPP) with GMX1778 (Fig. 2C–E; Supplementary Fig. S6). We thus compared the effects of treating PaTu8988T cells with vehicle control, a fixed subtherapeutic dose of GMX1778 [2 nmol/L; ∼5-fold below the GMX1778 EC_50_ value in control shGFP-expressing PaTu8988t cells (Fig. 2A)], increasing amounts of 4-Cl-HIPP, or GMX1778 + 4-Cl-HIPP combination in a colony-forming assay. Neither drug alone exhibited any substantial antiproliferative effect (see wells 1 and 5, Fig. 2C and D), but even the lowest dose of 4-Cl-HIPP (62.5 µmol/L) when combined with 2 nmol/L GMX1778 yielded substantially enhanced growth inhibition (see well 6, Fig. 2C and D) such that the calculated GI_50_ for 4-Cl-HIPP in PaTu8988T cells decreased >8-fold from ≫250 to 34 µmol/L (Fig. 2E). Moreover, two other PDAC cell lines demonstrated the same pattern of cooperativity of growth inhibition between GMX1778 and 4-Cl-HIPP as seen with PaTu8988T, where no inhibition was seen with vehicle, GMX1778 or 4-Cl-HIPP alone, but the combination of subtherapeutic GMX1778 (3 nmol/L) with 4-Cl-HIPP (250 µmol/L) caused statistically significant inhibition of growth that varied from 40% to 80% depending on the cell line (Supplementary Fig. S6). Untransformed HPNE cells were not inhibited by either drug alone or the combination (Supplementary Fig. S6), and though we observed an apparent approximately 50% growth inhibition of AsPC1 cells treated with the combination, the result did not achieve statistical significance. Notably, per TCGA, AsPC1 cells exhibit amplification at the NAPRT genetic locus (25), suggesting higher activity of the PH pathway is driving alternate NAD synthesis in these cells. Thus, NAMPT and CtBP inhibitors exert highly cooperative cancer-selective antiproliferative effects in certain PDAC cell lines, but not in untransformed pancreatic ductal cells or PDAC cells where alternative NAD synthesis pathways may be active.

### On-target Disruption of CtBP Oligomerization and Interaction with Transcriptional Corepressors by Combined NAMPT/CtBP Inhibition

**FIGURE 3:**
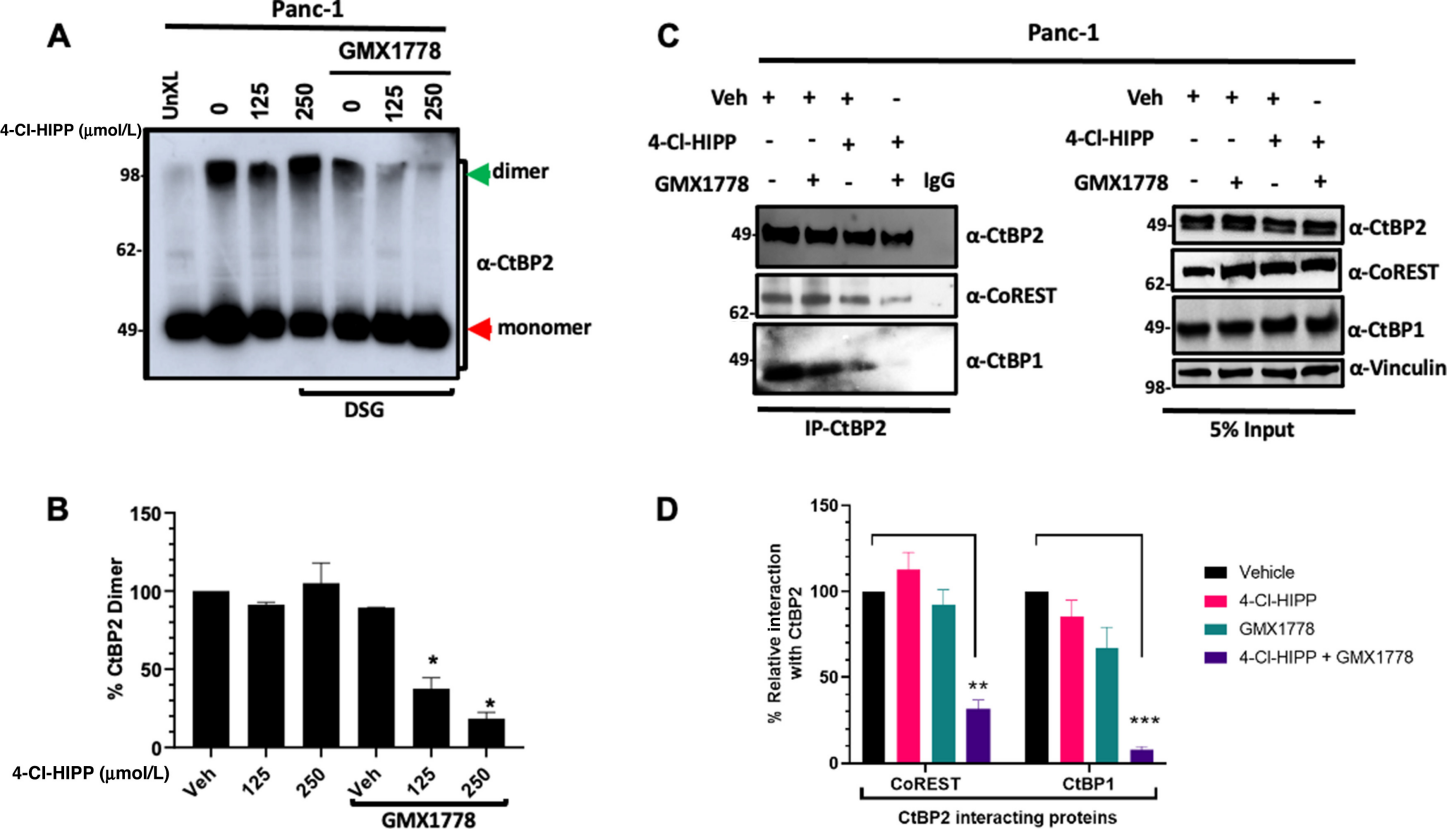
Combined NAMPT/CtBP inhibition attenuates physiologic CtBP dimerization and interaction with transcriptional coregulators. **A,** Panc-1 cells were treated with Vehicle (lanes 2–4) or 10 nmol/L GMX1778 (lanes 5–7) for 24 hours, followed by addition of Vehicle (0) or indicated concentrations of 4-Cl-HIPP for 48 hours, followed by incubating live cells in 0.25 µmol/L DSG and CtBP2 immunoblotting of cross-linked cell lysates. Results of a representative experiment from three independent experiments are shown. **B,** Densitometric quantitation of immunoblot in A. *P* values were determined by Student *t* test. **C,** Panc-1 cells were treated with Vehicle (Veh) or 10 nmol/L GMX1778 for 24 hours followed by addition of Vehicle or 250 µmol/L of 4-Cl-HIPP for 48 hours. Lysates of treated cells were then IPd with anti-CtBP2 antibody or control IgG (last lane) and IPs immunoblotted with CtBP1 or CoREST antibodies. Immunoblots of the input lysates are shown in the bottom. Vinculin was used as a loading control. Results of a representative experiment from three independent experiments are shown. **D,** Densitometric quantitation of immunoblot in C. *P* values were calculated using one-way ANOVA using Tukey *post hoc* correction. Error bars indicate ±1 SD. *, *P* < 0.05; **, *P* < 0.01; ***, *P* < 0.005 for comparison with vehicle-only treatment.

CtBP's oncogenic transcriptional activities require oligomer formation, and we previously reported that CtBP's oligomeric structure is disrupted *in vivo* by 4-Cl-HIPP, albeit inefficiently (10). To demonstrate that combined CtBP/NAMPT inhibition exhibits efficient on-target inhibition of CtBP oligomer formation in PDAC cells, we assayed CtBP dimerization using an *in situ* cross-linking assay (Fig. 3A; ref. 10). Panc-1 or PaTu8988T cells were treated with vehicle control, GMX1778 (10 nmol/L), increasing concentrations of 4-Cl-HIPP, or the combination of GMX1778 and 4-Cl-HIPP, and CtBP dimers were cross-linked by treatment of live cells with the chemical cross-linker disuccinimidyl glutarate, followed by immunoblotting of cross-linked cell lysates with CtBP1 or CtBP2 antibody to assay the abundance of CtBP dimers versus monomers (Fig. 3A and B; Supplementary Fig. S7). As seen in proliferation assays, we found that the ability of CtBP to form dimers was not reduced by 4-Cl-HIPP alone at concentrations tested, and GMX1778 alone caused observable <50% reductions in cross-linked CtBP1/2 dimers in the tested cell lines that did not achieve statistical significance. However, the combination of 4-Cl-HIPP and GMX1778 robustly disrupted CtBP1 and CtBP2 dimerization in Panc-1 cells (60% and 85% reduction relative to vehicle-only treatment and 45% and 85% reduction relative to GMX1778-only treatment, for CtBP1 and CtBP2, respectively) and in PaTu8988T cells (75% and 90% reduction relative to vehicle-only treatment and 50% and 90% reduction relative to GMX1778-only treatment, for CtBP1 and CtBP2, respectively; Fig. 3A and B; Supplementary Fig. S7).

Though the *in situ* cross-linking assay carries the advantage of freezing CtBP oligomeric structure within the living cell, providing “real time” readout of dimerization, it cannot distinguish CtBP1/2 homodimers from transcriptionally active CtBP1/2 heterodimers (26). We thus complemented the cross-linking assay with a co-IP assay to specifically examine the impact of combined CtBP/NAMPT inhibition on formation of CtBP1/2 heterodimers in Panc-1 and PaTu8988T cells (Fig. 3C and D; Supplementary Figs. S8 and S9). Indeed, CtBP2 IPs of lysates of Panc-1 cells that had been treated with vehicle, 4-Cl-HIPP, GMX1778, or the combination and immunoblotted for CtBP1 and CtBP2, revealed that the CtBPi/NAMPTi combination inhibited CtBP1/2 heterodimer formation by 90% (*P* < 0.001), while only minimal inhibition of heterodimer formation was observed with either drug alone (15% and 30%, respectively; Fig. 3C and D), with similar results observed in PaTu8988T cells (Supplementary Fig. S9A and S9B). In contrast, and unexpectedly, performing the cognate IP with CtBP1 antibody using the same cells and drug treatments, revealed no significant change in complexation of CtBP1 with CtBP2 under any condition (Supplementary Figs. S8, S9C, and S9D), which may be due to either extreme differences in absolute stoichiometry of CtBP1 and CtBP2 in the cell or the known dual localization of CtBP1 to cytoplasm and nucleus and known cellular variation in NAD metabolic pools and subcellular NAD concentrations (6, 27).

To further probe the impact of combined NAMPT/CtBP inhibition on oncogenic CtBP transcriptional function, we determined whether NAMPT/CtBP inhibition might disrupt active CtBP transcriptional repressor complexes (28). We previously established that CtBP2 interaction with the CoREST component of the CtBP transcriptional supercomplex could be disrupted using higher doses (>500 µmol/L) of 4-Cl-HIPP (10). We thus inquired whether 4-Cl-HIPP at lower pharmacologically relevant concentrations in combination with GMX1778 could more potently disrupt the CtBP/CoREST complex in Panc-1 (Fig. 3C and D) or PaTu8988T cells (Supplementary Fig. S9A and S9B). Indeed, the 4-Cl-HIPP/GMX1778 combination potently inhibited CtBP2 interaction with CoREST by 70% (*P* < 0.01) in Panc-1 cells (Fig. 3C and D) and by 60% in PaTu8988T cells (*P* < 0.05; Supplementary Fig. S9A and S9B), while neither drug alone had any significant effect. Conversely, and in alignment with the lack of NAMPTi/CtBPi effect when CtBP1–CtBP2 interaction was interrogated via CtBP1 IP (Supplementary Figs. S8, S9C, and S9D), the binding of CoREST to CtBP1 as assayed by CtBP1 IP was likewise not affected by GMX1778/4-Cl-HIPP, singly or in combination, in either Panc-1 (Supplementary Fig. S8) or PaTu8988T cells (Supplementary Fig. S9C and S9D). Thus, when assayed by CtBP2 IP, the 4-Cl-HIPP/GMX1778 combination exerts on-target inhibition of both CtBP hetero-oligomerization and interaction with CoREST, both of which are prerequisites for CtBP's oncogenic transcriptional functions (6, 8, 10), while the lack of observed effect when assayed by CtBP1 IP may be due to factors such as stoichiometric or subcellular localization that will require further study.

### NAMPTi/CtBPi Combination Blocks CtBP Oncogenic Transcriptional Regulation

**FIGURE 4:**
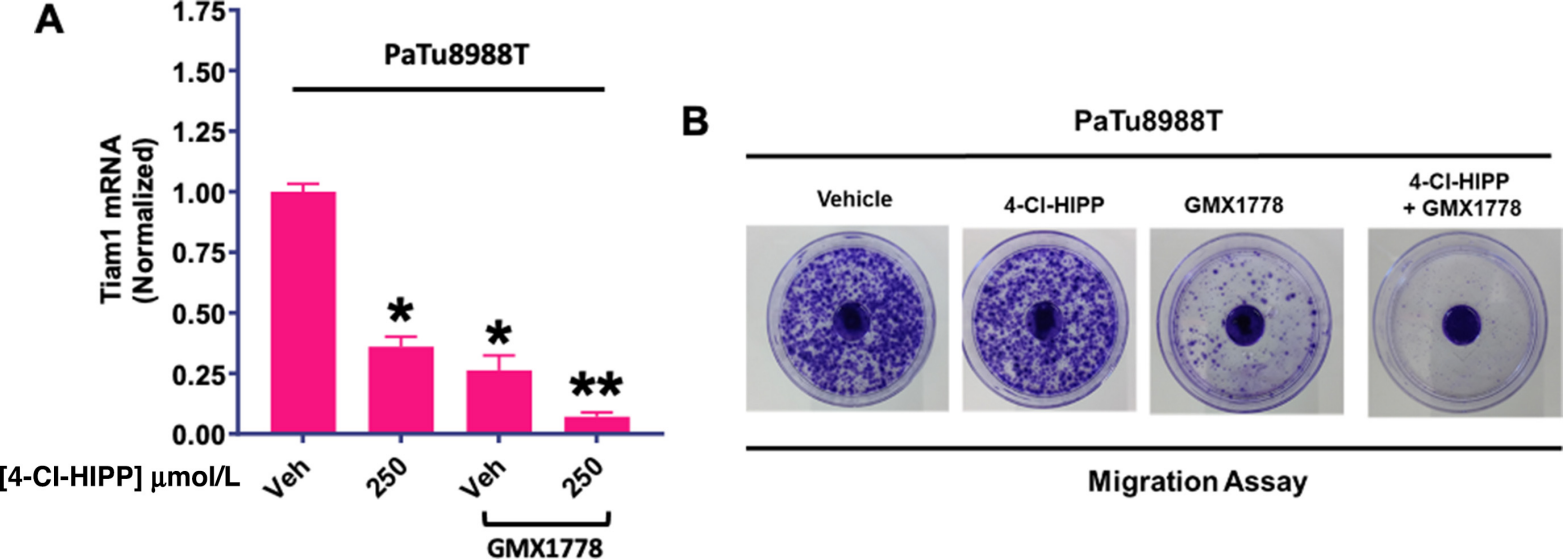
Combined NAMPT/CtBP inhibition potently represses CtBP transcriptional regulation and inhibits cellular motility. **A,** qPCR analysis of *TIAM1* (CtBP target gene) mRNA expression after indicated treatments for 24 hours in PaTu8988T cells. The calculation was performed using the ΔΔCt approach. *P* values were determined by Student *t* test. Error bars indicate ±1 SD. *, *P* < 0.05; **, *P* < 0.01 for comparison with vehicle-only treatment. **B,** Patu8988T cells were seeded into a glass bottom cell culture dish, treated with Vehicle or 2 nmol/L GMX1778 for 24 hours followed by addition of Vehicle or 250 µmol/L of 4-Cl-HIPP for 48 hours and then stained with crystal violet to detect cells that had migrated from the central groove toward the periphery of the dish. Results of a representative experiment from three independent experiments are shown.

To further validate that combined pharmacologic CtBP/NAMPT inhibition was acting mechanistically on target, we assayed the effects of 4-Cl-HIPP and GMX1778, alone or in combination, in a functional assay reflective of CtBP's archetypal oncogenic activities. We have already shown that genetic depletion of CtBP1 in combination with GMX1778 led to robust downregulation of expression of the CtBP transcriptional target Tiam1 (Fig. 2B). We thus treated PaTu8988T cells with vehicle control, or increasing concentrations of 4-Cl-HIPP, GMX1778, or the combination, and assayed *TIAM1* mRNA levels by qPCR (Fig. 4A). Though both 4-Cl-HIPP and GMX1778 each exhibited significant attenuation of *TIAM1* mRNA expression (65%–70% reduction; *P* < 0.05), the combination exhibited robust, nearly ablative reduction (90%; *P* < 0.01) of Tiam1 expression (ref. 14; Fig. 4A).

### NAMPTi/CtBPi Combination Blocks CtBP-mediated Cell Migration

A well-known oncogenic function of CtBP is to drive cancer cell migration and invasion in *in vitro* assays that correlates with its known activity driving metastasis in *in vivo* tumor models (6, 13, 14). To further validate on-target functional inhibition of CtBP oncogenic activities by the 4-Cl-HIPP/GMX1778 combination relevant to tumor progression, we studied how combined CtBP/NAMPT inhibition impacts PDAC cell migration using a modified wound-healing assay (Fig. 4B). PaTu8988T cells were plated in the groove of a glass-bottom cell culture dish and then treated with vehicle or GMX1778 for 24 hours, followed by addition of vehicle or 250 µmol/L of 4-Cl-HIPP for 48 hours, and then stained with crystal violet (Fig. 4B). As seen in many of the other functional assays, neither vehicle nor 4-Cl-HIPP, alone, impaired migration (Fig. 4B). In contrast, cells treated with GMX1778 demonstrated obvious migratory inhibition, while the combination of 4-Cl-HIPP and GMX1778 abrogated migration entirely (Fig. 4B), indicating that the drug combination can not only impair PDAC cell growth, but also attenuates PDAC cell motility/migration, which is importantly associated with PDAC tumor progression and metastatic potential (29).

### The 4-Cl-HIPP/GMX1778 Combination Attenuates Growth of PDAC Xenografts

**FIGURE 5:**
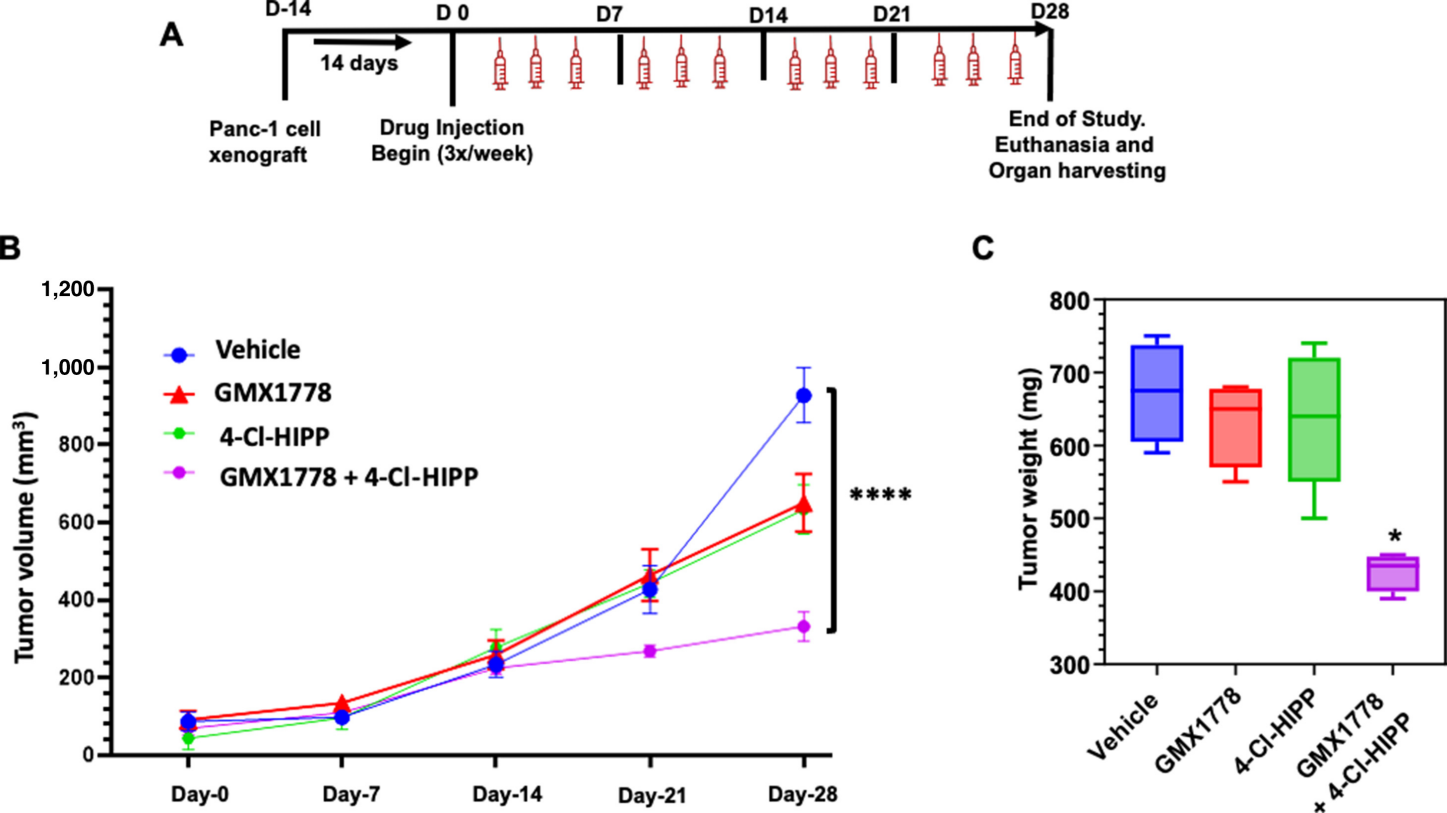
Combined NAMPT/CtBP inhibition abrogates growth of PDAC xenografts *in vivo*. **A,** Four cohorts of 5 NSG mice each were subcutaneously xenografted with 1 × 10^6^ Panc-1 cells and after 2 weeks of tumor growth, mice were treated three times per week for 4 weeks (see inset) by intraperitoneal injection of: (i) Vehicle (100 mmol/L NaHCO_3_); (ii) 4-Cl-HIPP (100 mg/kg); (iii) GMX1778 (30 mg/kg); or (iv) the combination of 4-Cl-HIPP and GMX1778. **B,** Tumor volume (mm^3^) was measured weekly using calipers and *P* values were calculated using multiple unpaired Student *t* test. Error bars indicate ±1 SD. ****, *P* < 0.0001 for comparison with vehicle-only treatment. **C,** Mean tumor weights determined at necropsy at the end of experiment on day 28 and *P* values were calculated using multiple unpaired Student *t* test. Error bars indicate ±1 SD. *, *P* < 0.05 for comparison with vehicle-only treatment.

Although CtBP and NAMPT inhibitors have demonstrated preclinical efficacy in PDAC models independently (13, 30, 31), our *in vitro* data would indicate that the rational combination of these agents will be substantially more efficacious, and provide a potential pathway to further therapeutic development. We therefore tested the *in vivo* efficacy of the NAMPTi/CtBPi combination by implanting Panc-1 cells in the flanks of immune-deficient NSG mice and treated them with vehicle, 4-Cl-HIPP [100 mg/kg (13)], GMX1778 [30 mg/kg (32)] or the combination, three times per week for 4 weeks or until humane endpoint (Fig. 5A). Remarkably, the mice treated with either agent alone, or in combination, exhibited no observable ill effects during the study, and the weight of the mice in all four cohorts remained unchanged throughout the period of treatment (Supplementary Fig. S10). However, examination of tumor volumes and weights after euthanasia at day 28 demonstrated that the combination treatment of 4-Cl-HIPP and GMX1778 significantly outperformed either monotherapy, as each drug alone exhibited a modest 33% reduction in tumor volume relative to vehicle treatment, but <10% effect on tumor weight, with neither difference achieving statistical significance (Fig. 5B and C). In contrast, the GMX1778/4-Cl-HIPP combination suppressed tumor volume by 67% and tumor weight by approximately 40% relative to vehicle treatment (*P* < 0.0001 and *P* < 0.05, respectively; Fig. 5B and C). The ability of the 4-Cl-HIPP/GMX1778 combination to effectively limit PDAC cell growth *in vitro* and tumor growth *in vivo* thus supports our hypothesis that combining CtBP inhibition with NAD depletion could be an effective and well-tolerated therapeutic strategy in human PDAC.

## Discussion

In this study, we have investigated the effect of inhibiting NAMPT-regulated salvage pathway NAD biosynthesis in PDAC cells and tumors using the NAMPTi GMX1778 as a means of sensitizing PDAC cells to genetic or pharmacologic inhibition of CtBP. We demonstrate that GMX1778 dramatically increased 4-Cl-HIPP effectiveness, decreasing its GI_50_ for PDAC cell lines 5- to 10-fold. The combination of NAMPT and CtBP inhibitors also cooperatively modulated CtBP's coregulatory activities and effectively attenuated tumor growth in a mouse xenograft model. Complementing and validating the pharmacologic effects of combined NAMPT/CtBP inhibition, CtBP depletion via RNAi in combination with NAMPTi mimicked the effects of inhibitor combination treatments.

Poor outcomes in PDAC treatment are reflective of the reliance on standard chemotherapy, and the ongoing lack of any novel efficacious therapeutic modalities, such as immunotherapy, which has exponentially improved survival in other refractory cancers. The enormous unmet needs in PDAC therapy are further highlighted by the poor outcomes observed even in earliest-stage disease (5-year survival after complete resection of a small stage I tumor is <50%), again due to the lack of effective systemic therapies that can be leveraged in the adjuvant setting. Our work over the past decade has amply demonstrated the critical dependency of many refractory solid tumors, including PDAC, on CtBP as a master transcriptional regulator (13, 14, 33), which has prompted our intensive effort to identify small-molecule inhibitors of the CtBP dehydrogenase catalytic domain (15). As it has become clear that CtBP oncogenic functions are critically dependent on NAD-driven oligomeric assembly (6, 8, 11, 20), we hypothesized that the antineoplastic efficacy of CtBP inhibitors would be greatly enhanced when used in combination with agents that disrupt NAD biosynthesis, and thus evaluated NAMPT inhibition in this work as a tool to lower cellular NAD levels as a means to enhance the cell inhibitory efficacy of CtBP inhibition (Figs. 1C, D, and 2; Supplementary Fig. S6).

CtBP oligomeric assembly and dehydrogenase activity follows a compulsory-order mechanism, binding to NAD first, followed by its substrate, MTOB (6). Furthermore, MTOB exhibited inhibitory activity when used in excess over NAD concentration by competing for the active site, acting as a substrate inhibitor (7). On the basis of this observation, several CtBP dehydrogenase active-site inhibitors were developed, including 4-Cl-HIPP, which was among the most potent analogs of the first-generation inhibitor and chemical scaffold, HIPP (15). Previously, we demonstrated that 4-Cl-HIPP inhibited CtBP oligomerization despite addition of NAD(H) (10), supporting our hypothesis that nucleotide binding to CtBP leads to its domain closure, wherein HIPP derivatives and NAD remain trapped in an inactive abortive ternary complex (34). To that end, we envisioned that a NAMPTi could reduce cellular levels of NAD and thus facilitate access of 4-Cl-HIPP to the CtBP dehydrogenase active site. As CtBP's oligomerization is central to its oncogenic activities and is facilitated by incorporation of NAD into its ternary structure (6), we show that the most efficacious strategy to disrupt CtBP dimers and interaction with transcription complexes is to simultaneously lower NAD levels via NAMPT inhibition and inhibit CtBP's dehydrogenase with a substrate competitive inhibitor such as 4-Cl-HIPP (Fig. 3; Supplementary Figs. S7 and S9). As CtBP oligomerization is a critical mechanistic link between chemical inhibition and functional inactivation, we envision that a tumor cell CtBP dimerization assay could serve as a companion pharmacodynamic biomarker to the dual NAMPT/CtBP inhibition therapeutic strategy.

Though in this work, we employ NAMPT inhibition as a strategy to enhance CtBP inhibition, NAMPT has been extensively pursued as a cancer therapeutic target, as certain tumors exhibit absolute dependency on the NAD salvage pathway due to the absence of functional *de novo* or PH pathways (4). However, NAMPT inhibitors have exhibited limited clinical success, due to both lack of robust efficacy as single agents, as well as dose-limiting toxicities (5). Indeed, PDAC cells are relatively resistant toward NAMPT inhibition as a single modality therapy, likely due to activity of complementing NAD biosynthetic pathways, such as the PH pathway, where the gene for the rate-limiting enzyme NAPRT is frequently amplified (4). Despite the lack of efficacy of NAMPT inhibitors as single agents in PDAC, we have successfully leveraged NAMPT inhibition as a tool to lower cellular NAD levels to enhance the cell inhibitory efficacy of CtBP inhibition (Figs. 1C, D, and 2; Supplementary Fig. S6), based on CtBP's absolute dependence on NAD to drive its oligomerization and oncogenic transcriptional activities (6). Moreover, we demonstrate PDAC cell growth inhibition that can be induced by GMX1778 as a single agent was reflective of on-target on-pathway activity, as NA completely rescued GMX1778-induced growth inhibition (Supplementary Fig. S5).

Because of the known overlapping and unique functions of the CtBP1/2 paralogs (6), we evaluated the effect of NAD depletion and CtBP dehydrogenase inhibition on oligomerization, heterodimerization, and transcriptional complex formation of both CtBP1 and CtBP2. We also compared the effects of CtBP1 or 2 depletion by shRNA on cooperation with GMX1778 to suppress growth of PDAC cell lines. CtBP1 and CtBP2 are highly similar in structure and sequence, and also share many overlapping functions in oncogenic transcriptional regulation and mitotic regulation (6). Indeed, recent work supports CtBP1/2 heterodimers as the active oligomeric form for transcriptional regulation (35). However, key distinctions include the lack of NLS in CtBP1 and also specific spliced forms of each protein with unique biologic functions, such as Golgi assembly (CtBP1) and neuronal synapses (CtBP2-BARS isoform; refs. 6, 36). In this work, we observed similar overall effects of CtBP1 and CtBP2 depletion on sensitization of growth inhibition by GMX1778 in two separate PDAC cell lines (Fig. 2A), and CtBP1 and CtBP2 dimerization was similarly inhibited by combined GMX1778/4-Cl-HIPP treatment in both Panc-1 and PaTu8988T cells (Fig. 3A; Supplementary Fig. S7).

The major distinction we observed between CtBP1 and CtBP2 was in the effect of NAMPT/CtBP inhibition on protein complexes recovered in CtBP1 versus CtBP2 IPs, (Fig. 3B; Supplementary Figs. S8 and S9), where GMX1778/4-Cl-HIPP treatment consistently disrupted recovery of both CtBP1 and CoREST in CtBP2 IPs of Panc-1 or PaTu8988T lysates, while recovery of CtBP2 and CoREST in CtBP1 IPs was completely unaffected by NAMPT/CtBP inhibition. We speculate that this strikingly different outcome between the effect of NAMPT/CtBP inhibition on complexes retrieved by CtBP1 versus CtBP2 IPs reflect either the differential subcellular localization of CtBP1 versus CtBP2 [CtBP1 has significant cytoplasmic localization and NAD levels vary across cellular compartments (6, 27)] or stoichiometric differences in CtBP1 versus CtBP2 cellular abundances that result in differential effects of the inhibitor treatments.

Nevertheless, our functional interrogation of CtBP oncogenic activity after NAMPT/CtBP inhibition reveled robust inhibition of oncogenic transcriptional regulation of the TIAM 1 gene, as well as disruption of cellular migration, which requires active CtBP oncogenic transcriptional regulation (14). Thus, we hypothesize that the complexes isolated in CtBP2 IPs (which include a subpopulation of CtBP1) reflect oncogenically active components of the NAD/CtBP axis, while complexes retrieved by CtBP1 antibody may be inactive due to localization or other factors specifically present in those complexes that remain to be elucidated.

This work points toward a future therapeutic strategy for pancreatic cancer, though the concentration of 4-Cl-HIPP needed for preclinical efficacy is still higher than desirable for a clinical agent, even when in combination with NAD lowering therapy. We are currently working to enhance the cell penetrability of HIPP-class CtBP inhibitors by developing new prodrug analogs of 4-Cl-HIPP where the carboxyl moiety has been esterified to improve its cell permeability. As we note that AsPC1 cells, which were relatively resistant to NAMPT/CtBP inhibition (Supplementary Fig. S6), demonstrate genetic amplification of the *NAPRT* locus, and potential hyperactivity of the PH pathway of NAD synthesis, we are also exploring the possibility of inhibiting NAPRT and the PH pathway in combination with CtBP/NAMPT inhibition, to more effectively lower NAD levels in PDAC cells and tumors where hyperactivity of complementing NAD synthetic pathways may lessen the efficacy of NAD reduction via NAMPT inhibition alone.

## Supplementary Material

Supplementary Figure 1 Overview of NAD biosynthesis pathways: Metabolites are in green shaded squares, and enzymes are in shaded ovals.

Supplementary Figure 2 Mean CtBP1/2 and NAMPT protein abundance in PDAC cell lines relative to levels in h-TERT-HPNE (HPNE) cells as determined by densitometry of corresponding immunoblots in Figs. 1B (Panel A) and 1C (Panel B).

Supplementary Figure 3 Immunoblot analysis of CtBP1/2 levels in PaTu8988T and Suit2 cell lines expressing shCtBP1/2 vs. shGFP control used in Fig. 2A.

Supplementary Figure 4 Viability of A) PaTu8988T or B) Suit2 cell lines expressing shGFP, shCtBP1, or shCtBP2, treated with Vehicle (Veh)or increasing concentrations of GMX1778 for 72 h as measured by MTT assay.

Supplementary Figure 5 Representative experiment showing the viability of PaTu8988T cells pretreated with vehicle or 10 µM of nicotinic acid (NA) and then treated with Vehicle (Veh) or increasing concentrations of GMX1778 for 72 h as measured by MTT assay. B) EC50 values for GMX1778 derived from 2 independent repetitions of the experiment in A).

Supplementary Figure 6 A592 of solubilized crystal violet from colony-forming assays of hTERT-HPNE, Panc-1, AsPC1, and BxPC3 cells treated with Vehicle or GMX1778 (3 nM) for 24 h followed by Vehicle (0) or 4-Cl-HIPP (250 µM) treatment for 5 days (Panc-1) or 7 days (hTERT-HPNE, BxPC3, AsPC1).

Supplementary Figure 7 Panc-1 or PaTu8988T cells were treated with Vehicle (Veh) or 10 nM GMX1778 for 24 h followed by the addition of Vehicle or indicated concentrations of 4-Cl-HIPP for 48 h, followed by incubating live cells in 0.25 µM disuccinimidyl glutarate (DSG), and immunoblotting of crosslinked cell lysates for CtBP1 (Panc-1, PaTu8988T) or CtBP2 (PaTu8988T).

Supplementary Figure 8 Panc-1 cells were treated with Vehicle (Veh) or 10 nM GMX1778 for 24 h followed by addition of Vehicle or 250 µM of 4-Cl-HIPP for 48 h.

Supplementary Figure 9 PaTu8988T cells were treated with Vehicle or 10 nM GMX1778 for 24 h followed by the addition of Vehicle or 250 µM of 4-Cl-HIPP for 48 h.

Supplementary Figure 10 Mean weight of mice in each of the 4 treatment cohorts from the xenograft study in Fig. 5

Supplementary Table 1 KRAS /TP53 status, sex and tissue origin for cell lines used in the study.
