# Supplementary material for: Combined Targeting of NAD Biosynthesis and the NAD-dependent Transcription Factor C-terminal Binding Protein as a Promising Novel Therapy for Pancreatic Cancer
Source: Cancer Res Commun. 2023 Oct 4;3(10):2003–13. doi: 10.1158/2767-9764.CRC-22-0521 (PMC10549224; doi:10.1158/2767-9764.CRC-22-0521)
Supplement: Supplementary Figure 1 — Overview of NAD biosynthesis pathways: Metabolites are in green shaded squares, and enzymes are in shaded ovals. [file crc-22-0521-s01.pdf]

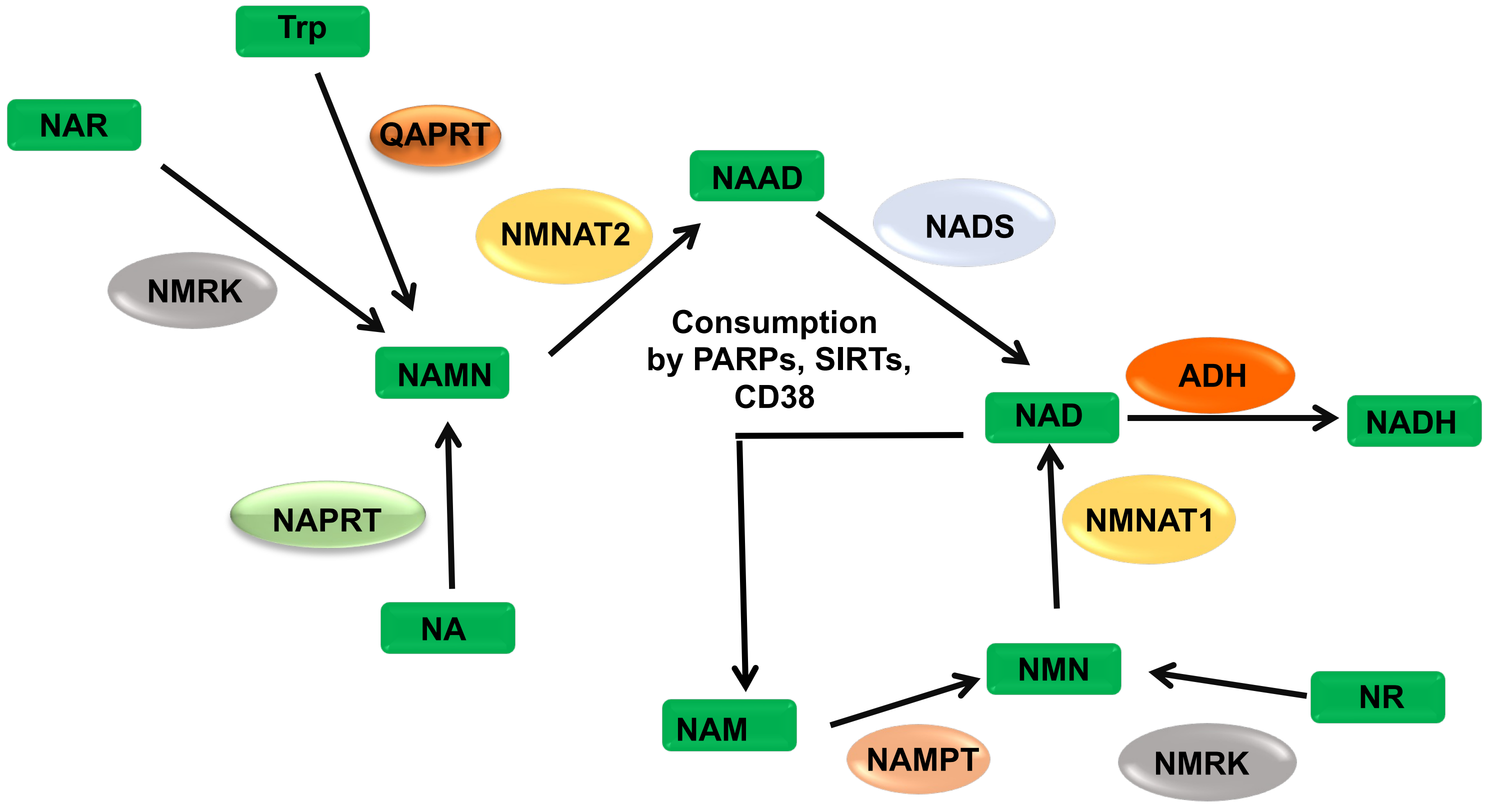


**Metabolites**

- NA- Nicotinic Acid
- NAMN- Nicotinic Acid Mononucleotide
- NAAD-Nicotinic Acid Adenine Dinucleotide
- NAR-Nicotinic Acid Riboside
- NAM- Nicotinamide
- NMN- Nicotinamide Mononucleotide
- NR-Nicotinamide Riboside
- NAD/ NADH- Nicotinamide Adenine Dinucleotide (Oxidized/Reduced form)

**Enzymes**

- NAMPT-Nicotinamide phosphoribosyltransferase
- NAPRT- Nicotinate phosphoribosyltransferase
- NMNAT-Nicotinamide mono-nucleotide adenylyltransferase
- NMRK-Nicotinamide adenine dinucleotide kinase
- NADS-NAD synthetase
- QAPRT-Quinolinate phosphoribosyltransferase
- ADH-Alcohol Dehydrogenase

**Supp. Fig. 1.** Overview of NAD biosynthesis pathways: Metabolites are in green shaded squares, and enzymes are in shaded ovals. NAD can be synthesized from NAM, NA, and their corresponding ribosides NR, NAR, and Trp. NAM is the preferred NAD precursor in humans and the product of the NAD salvage pathway for which NAMPT is the rate-limiting enzyme. The Preiss–Handler pathway utilizes NAPRT (rate-limiting enzyme; frequently amplified in PDAC), NMNAT, and NADS to synthesize NAD from diet-derived NA. QAPRT is the rate-limiting enzyme in the Trp-dependent *de novo* NAD synthesis pathway.
