# Supplementary material for: Combined Targeting of NAD Biosynthesis and the NAD-dependent Transcription Factor C-terminal Binding Protein as a Promising Novel Therapy for Pancreatic Cancer
Source: Cancer Res Commun. 2023 Oct 4;3(10):2003–13. doi: 10.1158/2767-9764.CRC-22-0521 (PMC10549224; doi:10.1158/2767-9764.CRC-22-0521)
Supplement: Supplementary Figure 2 — Mean CtBP1/2 and NAMPT protein abundance in PDAC cell lines relative to levels in h-TERT-HPNE (HPNE) cells as determined by densitometry of corresponding immunoblots in Figs. 1B (Panel A) and 1C (Panel B). [file crc-22-0521-s02.pdf]

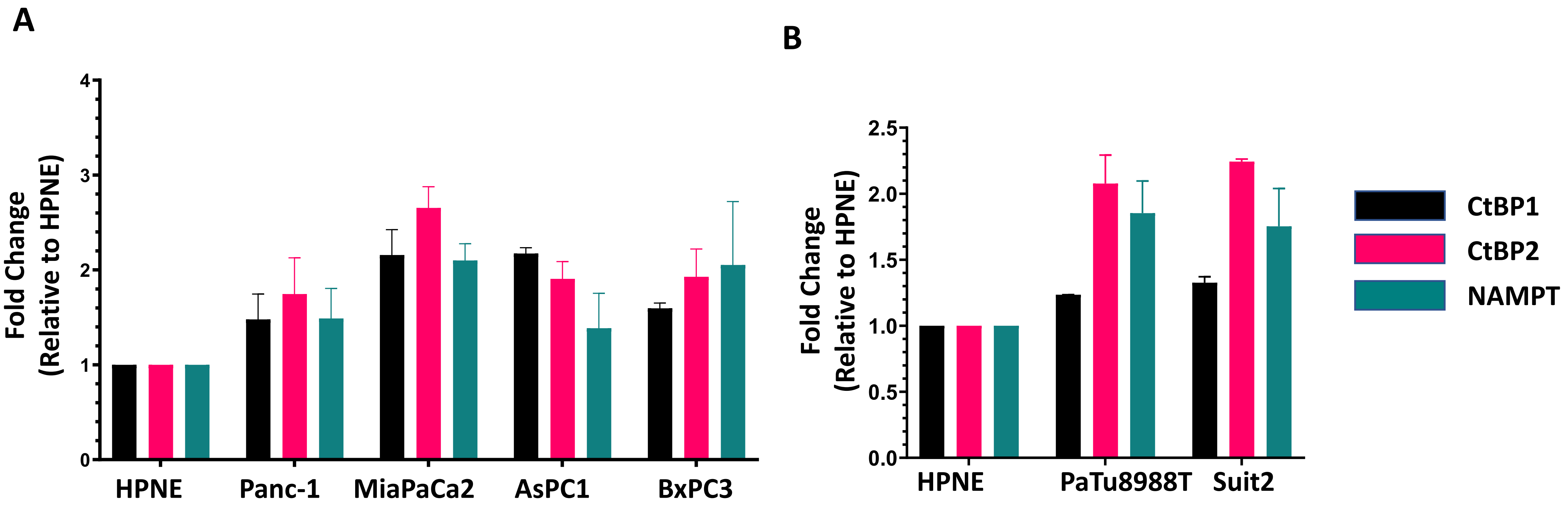

Supp. Fig. 2. Mean CtBP1/2 and NAMPT protein abundance in PDAC cell lines relative to levels in h-TERT-HPNE (HPNE) cells as determined by densitometry of corresponding immunoblots in Figs. **1B (Panel A)** and **1C (Panel B)**. Error bars indicate +/- 1 standard deviation. N=3 independent experiments.
