# Supplementary material for: Combined Targeting of NAD Biosynthesis and the NAD-dependent Transcription Factor C-terminal Binding Protein as a Promising Novel Therapy for Pancreatic Cancer
Source: Cancer Res Commun. 2023 Oct 4;3(10):2003–13. doi: 10.1158/2767-9764.CRC-22-0521 (PMC10549224; doi:10.1158/2767-9764.CRC-22-0521)
Supplement: Supplementary Figure 3 — Immunoblot analysis of CtBP1/2 levels in PaTu8988T and Suit2 cell lines expressing shCtBP1/2 vs. shGFP control used in Fig. 2A. [file crc-22-0521-s03.pdf]

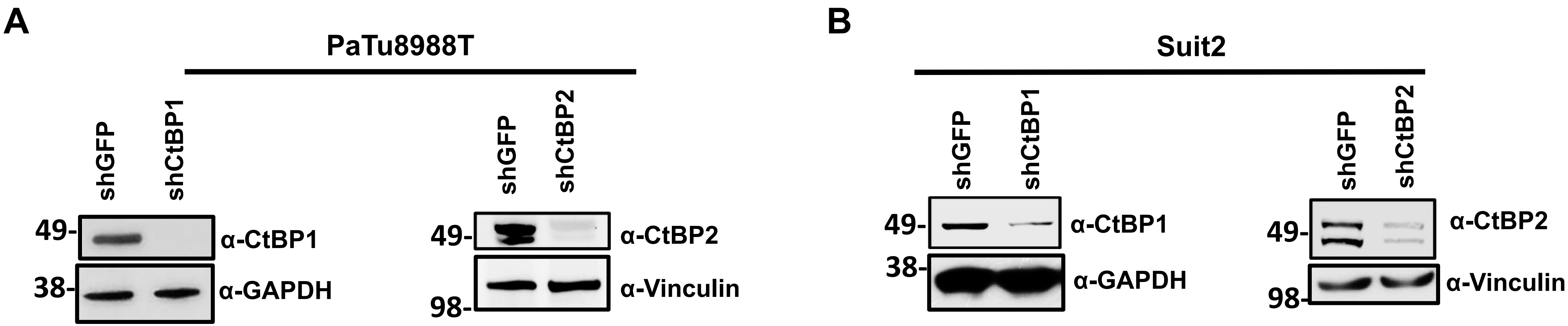

Supp. Fig. 3. Immunoblot analysis of CtBP1/2 levels in PaTu8988T and Suit2 cell lines expressing shCtBP1/2 vs. shGFP control used in **Fig. 2A**. GAPDH/Vinculin are loading controls.
