# Supplementary material for: Combined Targeting of NAD Biosynthesis and the NAD-dependent Transcription Factor C-terminal Binding Protein as a Promising Novel Therapy for Pancreatic Cancer
Source: Cancer Res Commun. 2023 Oct 4;3(10):2003–13. doi: 10.1158/2767-9764.CRC-22-0521 (PMC10549224; doi:10.1158/2767-9764.CRC-22-0521)
Supplement: Supplementary Figure 4 — Viability of A) PaTu8988T or B) Suit2 cell lines expressing shGFP, shCtBP1, or shCtBP2, treated with Vehicle (Veh)or increasing concentrations of GMX1778 for 72 h as measured by MTT assay. [file crc-22-0521-s04.pdf]

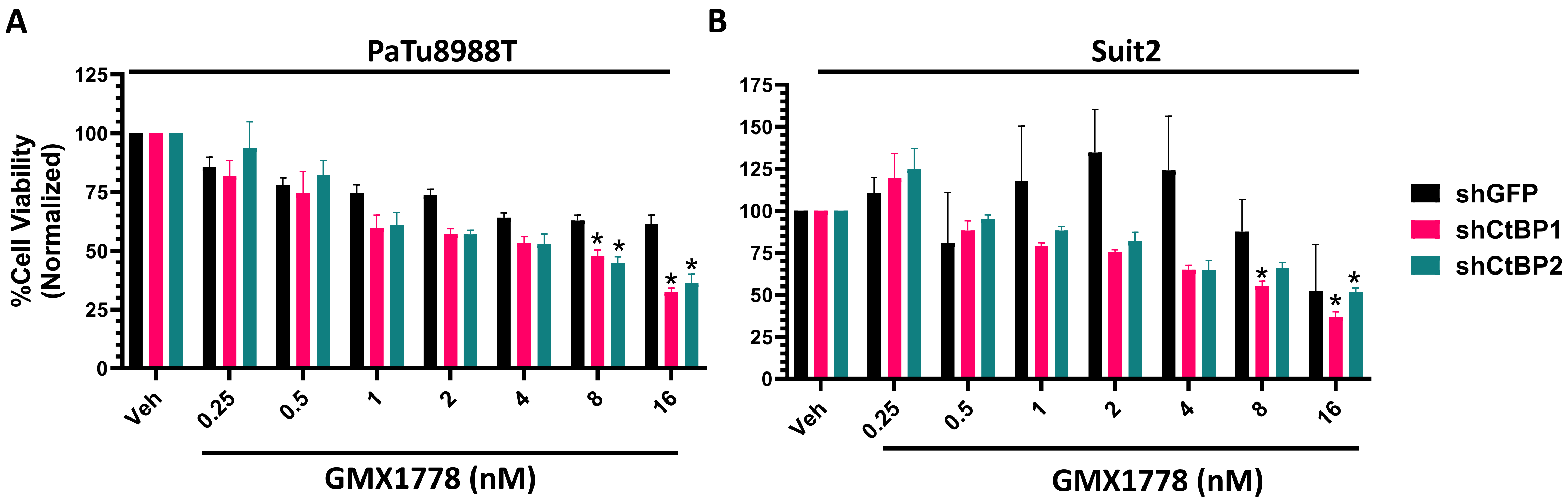

Supp. Fig. 4. Viability of **A)** PaTu8988T or **B)** Suit2 cell lines expressing shGFP, shCtBP1, or shCtBP2, treated with Vehicle (Veh) or increasing concentrations of GMX1778 for 72 h as measured by MTT assay. Bars indicate the viability of each cell type relative to the vehicle treatment. Error bars indicate +/- 1 standard deviation. N=3 independent experiments. p values were calculated relative to vehicle treatment using Student's t-test. *p<0.05.
