# Supplementary material for: Combined Targeting of NAD Biosynthesis and the NAD-dependent Transcription Factor C-terminal Binding Protein as a Promising Novel Therapy for Pancreatic Cancer
Source: Cancer Res Commun. 2023 Oct 4;3(10):2003–13. doi: 10.1158/2767-9764.CRC-22-0521 (PMC10549224; doi:10.1158/2767-9764.CRC-22-0521)
Supplement: Supplementary Figure 5 — Representative experiment showing the viability of PaTu8988T cells pretreated with vehicle or 10 µM of nicotinic acid (NA) and then treated with Vehicle (Veh) or increasing concentrations of GMX1778 for 72 h as measured by MTT assay. B) EC50 values for GMX1778 derived from 2 independent repetitions of the experiment in A). [file crc-22-0521-s05.pdf]

A

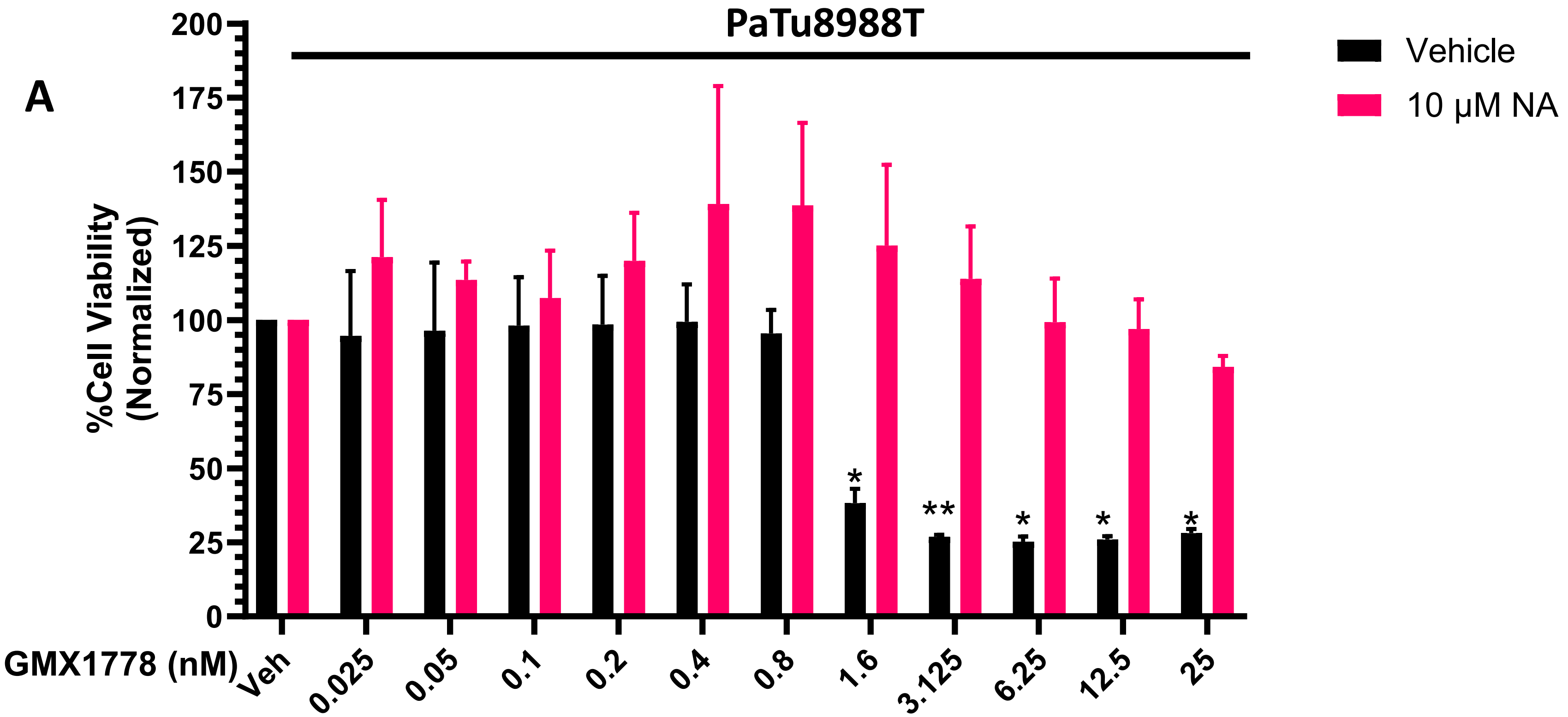

B

	EC_{50} GMX1778 (nM)			
**Cell Type**	**Experiment-1**		**Experiment-2**	
**PaTu8988T**	**Veh**	**NA**	**Veh**	**NA**
	**2.3**	**>>25#**	**4.4**	**>>25#**

Supp. Fig. 5. A) Representative experiment showing the viability of PaTu8988T cells pretreated with vehicle or 10 μM of nicotinic acid (NA) and then treated with Vehicle (Veh) or increasing concentrations of GMX1778 for 72 h as measured by MTT assay. **B**) EC_{50} values for GMX1778 derived from 2 independent repetitions of the experiment in **A)**. #Indicates EC_{50} was beyond the range of the titration and could not be determined. N=2 independent experiments. Error bars indicate +/- 1 standard deviation. p values were calculated relative to vehicle treatment using Student's t-test. *p<0.05; **p<0.01.
