# Supplementary material for: Combined Targeting of NAD Biosynthesis and the NAD-dependent Transcription Factor C-terminal Binding Protein as a Promising Novel Therapy for Pancreatic Cancer
Source: Cancer Res Commun. 2023 Oct 4;3(10):2003–13. doi: 10.1158/2767-9764.CRC-22-0521 (PMC10549224; doi:10.1158/2767-9764.CRC-22-0521)
Supplement: Supplementary Figure 6 — A592 of solubilized crystal violet from colony-forming assays of hTERT-HPNE, Panc-1, AsPC1, and BxPC3 cells treated with Vehicle or GMX1778 (3 nM) for 24 h followed by Vehicle (0) or 4-Cl-HIPP (250 µM) treatment for 5 days (Panc-1) or 7 days (hTERT-HPNE, BxPC3, AsPC1). [file crc-22-0521-s06.pdf]

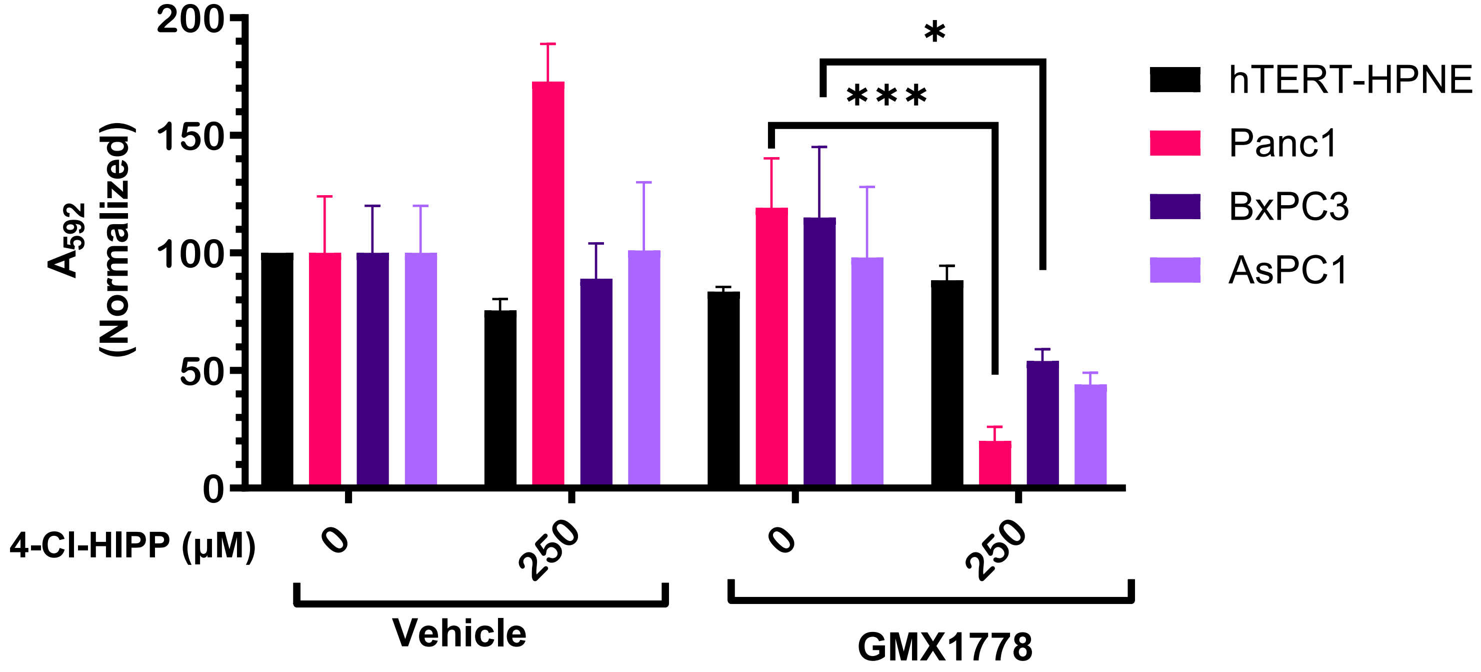

Supp. Fig. 6. A_{592} of solubilized crystal violet from colony-forming assays of hTERT-HPNE, Panc-1, AsPC1, and BxPC3 cells treated with Vehicle or GMX1778 (3 nM) for 24 h followed by Vehicle (0) or 4-Cl-HIPP (250 µM) treatment for 5 days (Panc-1) or 7 days (hTERT-HPNE, BxPC3, AsPC1). N=3 independent experiments. Error bars indicate +/- 1 standard deviation. *p<0.05; ***p<0.001 using Student's t-test for comparisons indicated by brackets.
