# Supplementary material for: Combined Targeting of NAD Biosynthesis and the NAD-dependent Transcription Factor C-terminal Binding Protein as a Promising Novel Therapy for Pancreatic Cancer
Source: Cancer Res Commun. 2023 Oct 4;3(10):2003–13. doi: 10.1158/2767-9764.CRC-22-0521 (PMC10549224; doi:10.1158/2767-9764.CRC-22-0521)
Supplement: Supplementary Figure 7 — Panc-1 or PaTu8988T cells were treated with Vehicle (Veh) or 10 nM GMX1778 for 24 h followed by the addition of Vehicle or indicated concentrations of 4-Cl-HIPP for 48 h, followed by incubating live cells in 0.25 µM disuccinimidyl glutarate (DSG), and immunoblotting of crosslinked cell lysates for CtBP1 (Panc-1, PaTu8988T) or CtBP2 (PaTu8988T). [file crc-22-0521-s07.pdf]

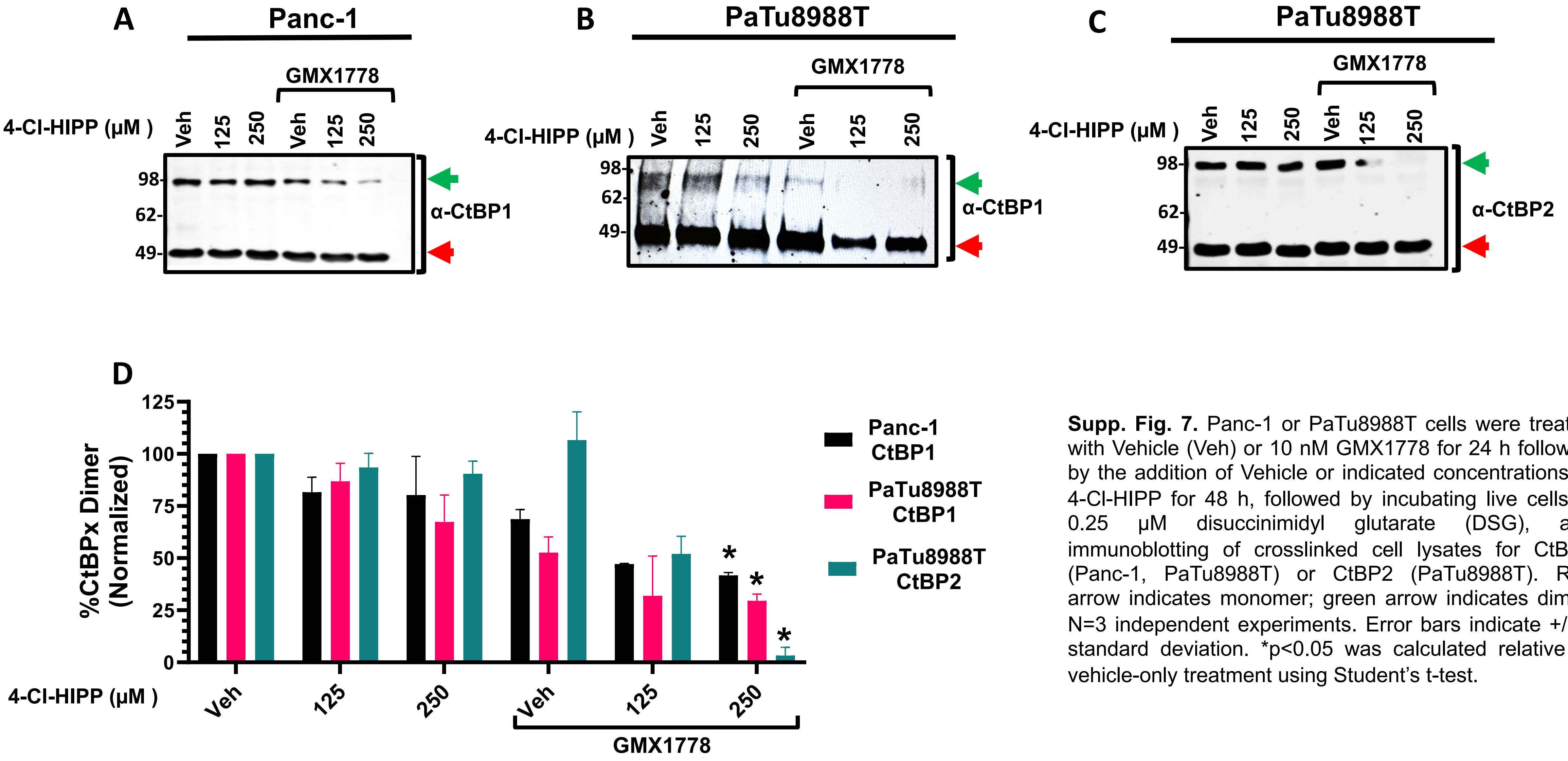

Supp. Fig. 7. Panc-1 or PaTu8988T cells were treated with Vehicle (Veh) or 10 nM GMX1778 for 24 h followed by the addition of Vehicle or indicated concentrations of 4-Cl-HIPP for 48 h, followed by incubating live cells in 0.25 μM disuccinimidyl glutarate (DSG), and immunoblotting of crosslinked cell lysates for CtBP1 (Panc-1, PaTu8988T) or CtBP2 (PaTu8988T). Red arrow indicates monomer; green arrow indicates dimer. N=3 independent experiments. Error bars indicate +/- 1 standard deviation. *p<0.05 was calculated relative to vehicle-only treatment using Student's t-test.
