# Supplementary material for: Combined Targeting of NAD Biosynthesis and the NAD-dependent Transcription Factor C-terminal Binding Protein as a Promising Novel Therapy for Pancreatic Cancer
Source: Cancer Res Commun. 2023 Oct 4;3(10):2003–13. doi: 10.1158/2767-9764.CRC-22-0521 (PMC10549224; doi:10.1158/2767-9764.CRC-22-0521)
Supplement: Supplementary Figure 8 — Panc-1 cells were treated with Vehicle (Veh) or 10 nM GMX1778 for 24 h followed by addition of Vehicle or 250 µM of 4-Cl-HIPP for 48 h. [file crc-22-0521-s08.pdf]

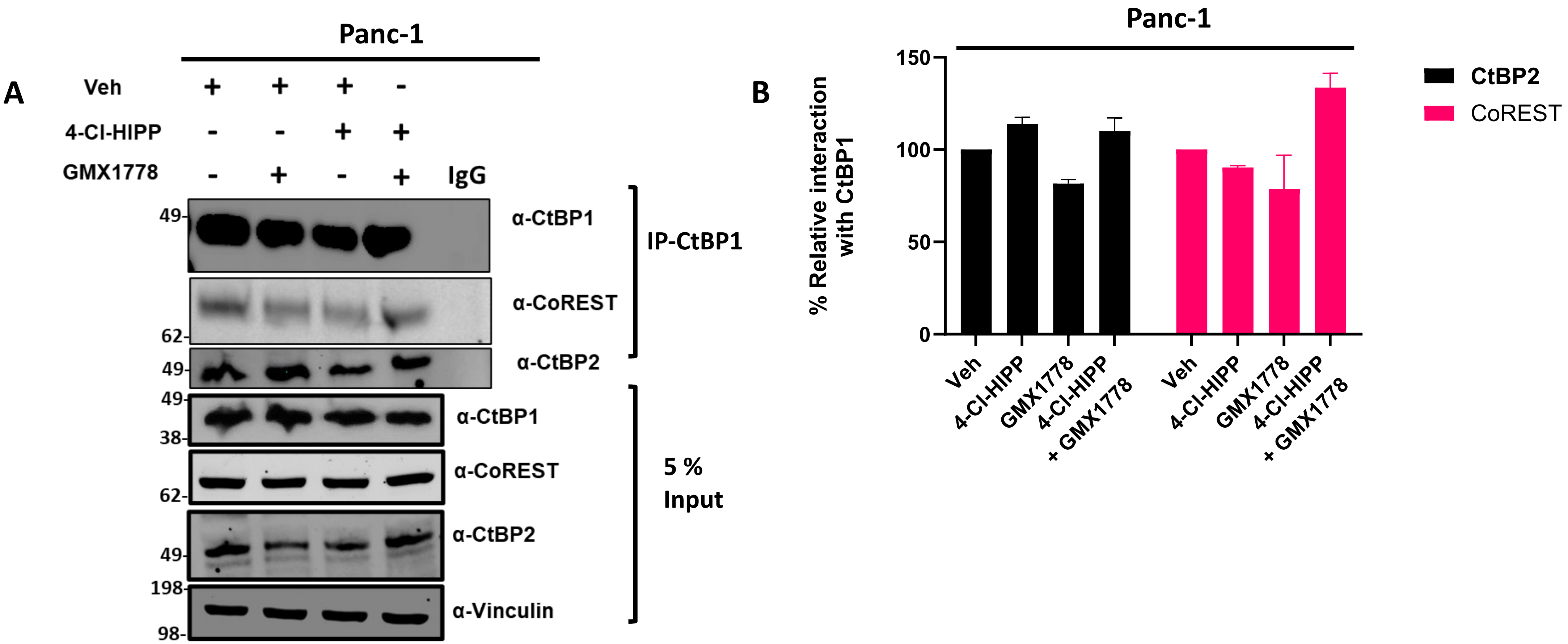

Supp. Fig. 8. Panc-1 cells were treated with Vehicle (Veh) or 10 nM GMX1778 for 24 h followed by addition of Vehicle or 250 μM of 4-Cl-HIPP for 48 h. Lysates of treated cells were then IP'd with anti-CtBP1 antibody or control IgG. **A)** Immunoblots of IPs and input lysates using CtBP2, CoREST, and vinculin (loading control) antibodies. **B)** Densitometric quantitation of CtBP1 IP immunoblots. N=3 independent experiments. Error bars indicate +/- 1 standard deviation.
