# Supplementary material for: Combined Targeting of NAD Biosynthesis and the NAD-dependent Transcription Factor C-terminal Binding Protein as a Promising Novel Therapy for Pancreatic Cancer
Source: Cancer Res Commun. 2023 Oct 4;3(10):2003–13. doi: 10.1158/2767-9764.CRC-22-0521 (PMC10549224; doi:10.1158/2767-9764.CRC-22-0521)
Supplement: Supplementary Figure 9 — PaTu8988T cells were treated with Vehicle or 10 nM GMX1778 for 24 h followed by the addition of Vehicle or 250 µM of 4-Cl-HIPP for 48 h. [file crc-22-0521-s09.pdf]

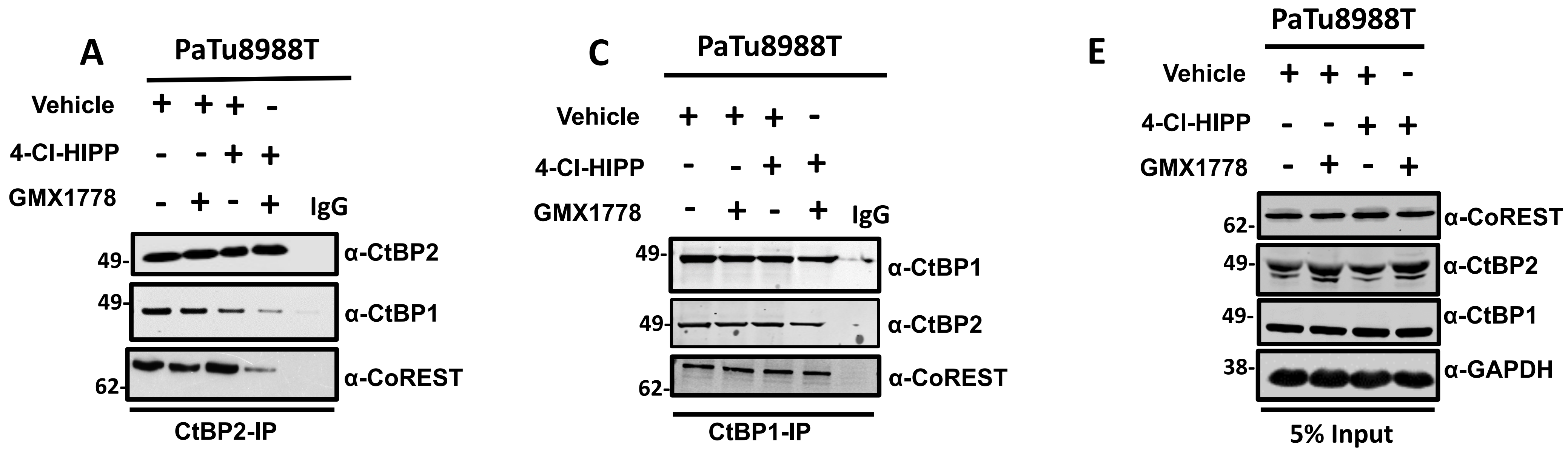

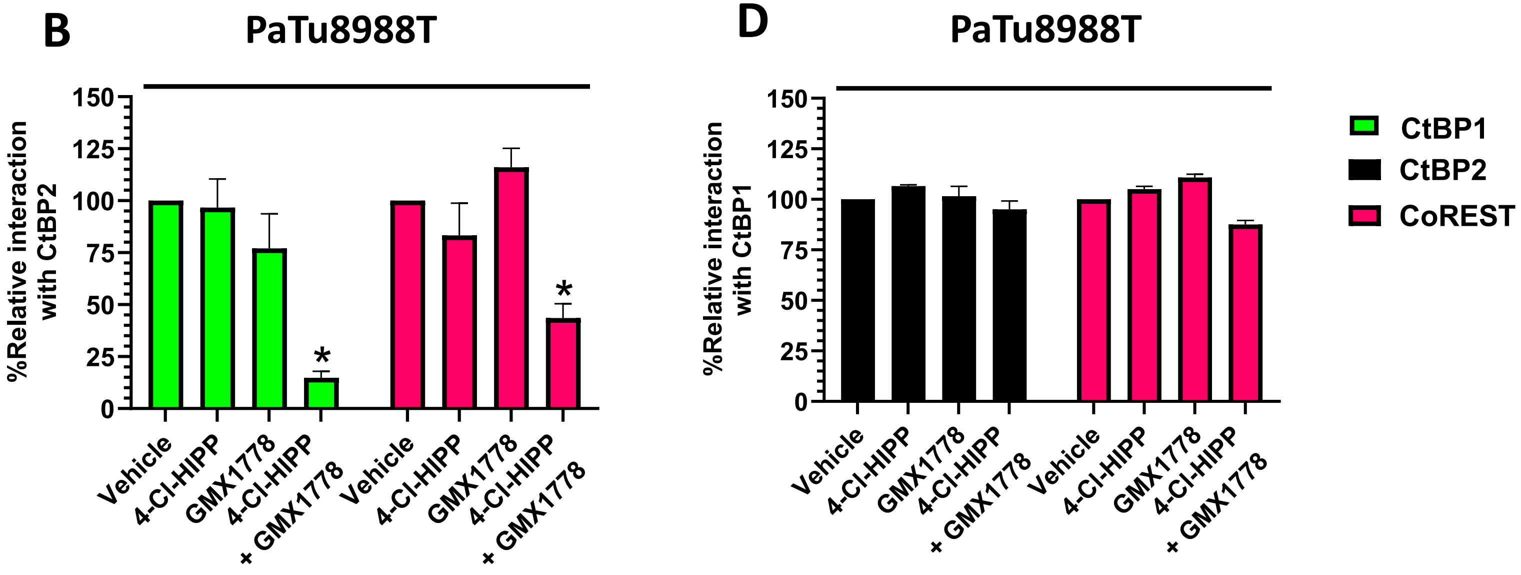

Supp. Fig. 9. PaTu8988T cells were treated with Vehicle or 10 nM GMX1778 for 24 h followed by the addition of Vehicle or 250 μM of 4-Cl-HIPP for 48 h. Lysates of treated cells were then IP'd with **A)** anti-CtBP2, **C)** anti-CtBP1 antibody, or control IgG (last lanes in **A)** and **C)**) and IPs immunoblotted with CtBP1, CtBP2, or CoREST antibodies. **B)** Densitometric quantitation of CtBP2 IP immunoblot in **A)**. **D)** Densitometric quantitation of CtBP1 IP immunoblot in **C)**. **E)** Immunoblots of the input lysates used in **A)** and **C)** with indicated antibodies including GAPDH (loading control). N=3 independent experiments. Error bars indicate +/- 1 standard deviation. *p<0.05 and was calculated relative to vehicle-only treatment using one-way ANOVA with Tukey's post-hoc correction.
