# Supplementary material for: Combined Targeting of NAD Biosynthesis and the NAD-dependent Transcription Factor C-terminal Binding Protein as a Promising Novel Therapy for Pancreatic Cancer
Source: Cancer Res Commun. 2023 Oct 4;3(10):2003–13. doi: 10.1158/2767-9764.CRC-22-0521 (PMC10549224; doi:10.1158/2767-9764.CRC-22-0521)
Supplement: Supplementary Figure 10 — Mean weight of mice in each of the 4 treatment cohorts from the xenograft study in Fig. 5 [file crc-22-0521-s10.pdf]

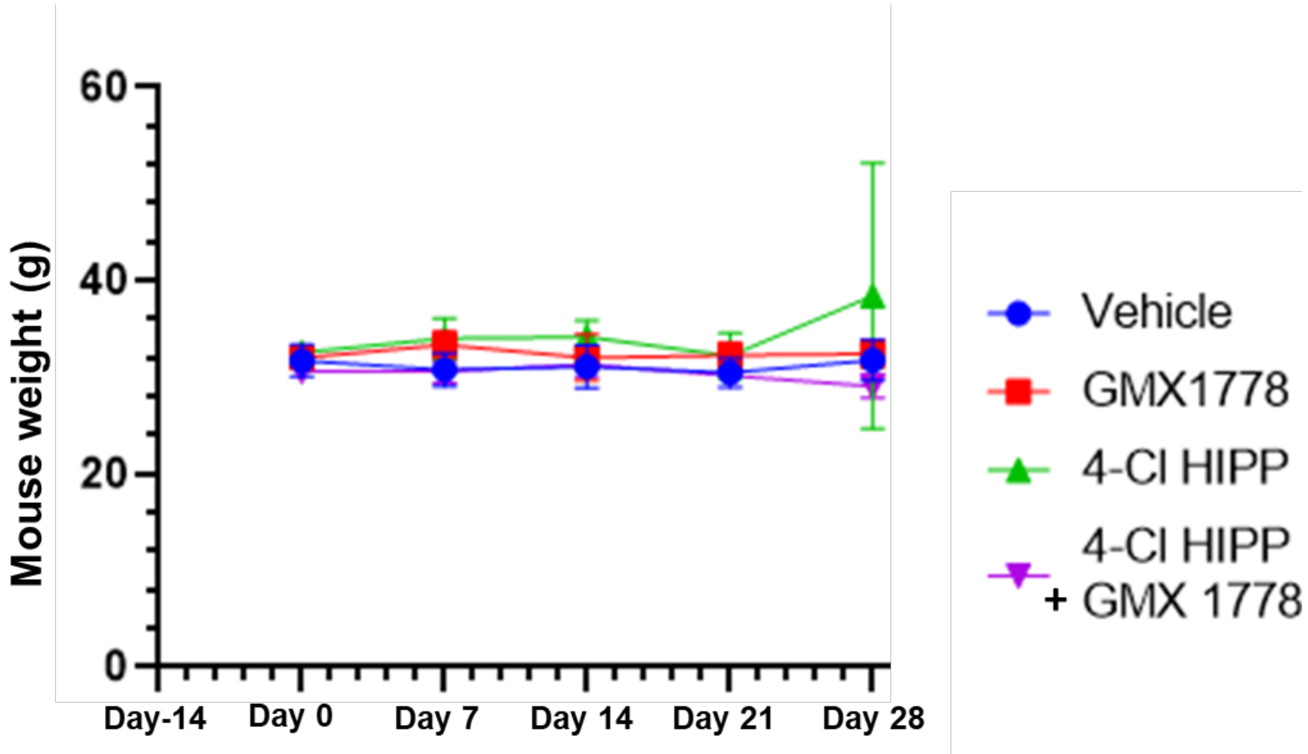

Supp. Fig. 10. Mean weight of mice in each of the 4 treatment cohorts from the xenograft study in **Fig. 5** was measured weekly during, and at the end of the study, on day 28. Error bars indicate +/- 1 standard deviation (N=5/cohort). Day -14 is the day tumor cells were injected into the flanks.
