# Supplementary material for: Combined Targeting of NAD Biosynthesis and the NAD-dependent Transcription Factor C-terminal Binding Protein as a Promising Novel Therapy for Pancreatic Cancer
Source: Cancer Res Commun. 2023 Oct 4;3(10):2003–13. doi: 10.1158/2767-9764.CRC-22-0521 (PMC10549224; doi:10.1158/2767-9764.CRC-22-0521)
Supplement: Supplementary Table 1 — KRAS /TP53 status, sex and tissue origin for cell lines used in the study. [file crc-22-0521-s11.pdf]

Cell name	KRAS	TP53	Gender	Tissue Origin
**PaTu8988T**	G12V	R282W	Female	Primary Tumor
**Suit2**	G12D	R273H	Male	Metastatic Site: Liver
**Panc-1**	G12D	R273H	Male	Primary Tumor
**MiaPaCa-2**	G12C	R248W	Male	Primary Tumor
**AsPc-1**	G12D	C35Afs*35	Female	Ascites
**BxPC3**	Wild-type	Y220C	Female	Primary Tumor
**hTERT-HPNE**	Wild-Type	Wild-Type	Male	Pancreas duct

Supp. Table 1. *KRAS* /*TP53* status, sex and tissue origin for cell lines used in the study.
